# Emerging Nanomaterials for Drinking Water Purification: A New Era of Water Treatment Technology

**DOI:** 10.3390/nano14211707

**Published:** 2024-10-25

**Authors:** Salma Elhenawy, Majeda Khraisheh, Fares AlMomani, Mohammad Al-Ghouti, Rengaraj Selvaraj, Ala’a Al-Muhtaseb

**Affiliations:** 1 Department of Chemical Engineering, College of Engineering, Qatar University, Doha 2713, Qatar; salma.elhenawy@qu.edu.qa (S.E.); falmomani@qu.edu.qa (F.A.); 2 Environmental Sciences Program, Department of Biological and Environmental Sciences, College of Arts and Sciences, Qatar University, Doha 2713, Qatar; mohammad.alghouti@qu.edu.qa; 3 Department of Chemistry, Sultan Qaboos University, Muscat 123, Oman; rengaraj@squ.edu.om; 4 Department of Petroleum and Chemical Engineering, Sultan Qaboos University, Muscat 123, Oman; almuhtasebala@gmail.com; 5 Sustainable Energy Research Centre, Sultan Qaboos University, Muscat 123, Oman

**Keywords:** nanomaterials, water purification, graphene, hydrogels, carbon nanotubes

## Abstract

The applications of nanotechnology in the field of water treatment are rapidly expanding and have harvested significant attention from researchers, governments, and industries across the globe. This great interest stems from the numerous benefits, properties, and capabilities that nanotechnology offers in addressing the ever-growing challenges related to water quality, availability, and sustainability. This review paper extensively studies the applications of several nanomaterials including: graphene and its derivative-based adsorbents, CNTs, TiO_2_ NPs, ZnO NPs, Ag NPs, Fe NPs, and membrane-based nanomaterials in the purification of drinking water. This, it is hoped, will provide the water treatment sector with efficient materials that can be applied successfully in the water purification process to help in addressing the worldwide water scarcity issue.

## 1. Introduction

Access to clean drinking water is a basic human right, but global water scarcity and pollution make it increasingly difficult. Although two-thirds of Earth’s surface is water, only 0.3% is freshwater available for the 8.1 billion global population [1,2]. Water scarcity threatens human activities as freshwater resources are depleted. Current water treatment systems are unable to meet growing demand, with 2 billion people lacking safe water and 1.2 billion people having only basic water services [3].

**Figure 1:**
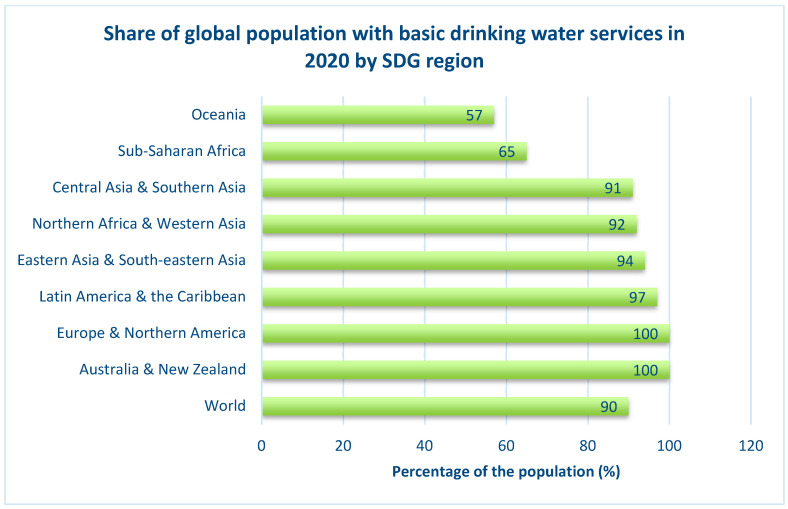
Estimated share of global population with basic drinking water services by SDG region in the year 2020. (Source: UNICEF).

By looking at Figure 1, it is clearly seen that Oceania and Africa are the regions with the least percentage availability of drinking water, with values of 57% and 65%, respectively, in the year 2020. This means that almost half of the population lack access to clean and safe drinking water in those regions. The main issues that currently face the water supply system and limit its function are population growth, global warming, and water pollution. In the year 2014, the World Health Organization (WHO) predicted that half of the world’s population would live in water-scarce areas by the year 2025 [4]. Hence, innovative, engineered, efficient water purification techniques are urgently required for the production of clean and safe drinking water.

Water purification technologies are gaining global attention as clean drinking water becomes scarce. Sustainable innovations are needed for cost-effective, energy-efficient solutions. Nanotechnology offers long-term potential by improving filtration materials, enhancing water quality, and reducing costs. Nanomaterials, engineered at the nanoscale (under 100 nanometers), play a key role in these advancements [5]. Nanomaterials, such as carbon nanotubes (CNTs) and graphene oxide (GO), have unique properties that make them effective in water purification. Their high porosity and reactivity allow them to capture various contaminants, including germs, organic pollutants, heavy metals, and viruses, making them valuable in filtration systems to address water scarcity challenges. [6]. In addition, nanomaterials play a crucial role in water disinfection processes. For example, silver nanoparticles have strong antibacterial capabilities that can successfully prevent the growth of pathogenic germs in water. Bhardwaj, et al. [7] mentioned in their study that silver-based porous nanocomposites (AgNCs) can efficiently kill 99–100% of bacteria and viruses from drinking water. Furthermore, titanium dioxide nanoparticles produce reactive oxygen species that kill the bacteria and viruses found in water when exposed to ultraviolet (UV) light [8]. The use of nanomaterials allows for effective, chemical-free water disinfection, avoiding harmful byproducts from traditional chlorination.

The scope of this review paper is to comprehensively examine the application of various nanomaterials in the purification of drinking water. The focus is on a range of materials, including graphene and its derivatives as water pollutant adsorbents, carbon nanotubes (CNTs), titanium dioxide (TiO_2_) nanoparticles, zinc oxide (ZnO) nanoparticles, silver (Ag) nanoparticles, iron (Fe) nanoparticles, and nanomaterials incorporated into membrane technologies for water purification. This paper aims to evaluate these materials for their effectiveness in removing a broad spectrum of water contaminants, including heavy metals, organic pollutants, pathogens, and emerging contaminants. By assessing the potential of these nanomaterials, this review intends to provide insights into their practical applications in large-scale water treatment systems. The ultimate goal is to support the water treatment industry by identifying efficient and scalable nanomaterial-based solutions that can contribute to addressing the global water crisis.

## 2. Drinking Water Sources

**Figure 2:**
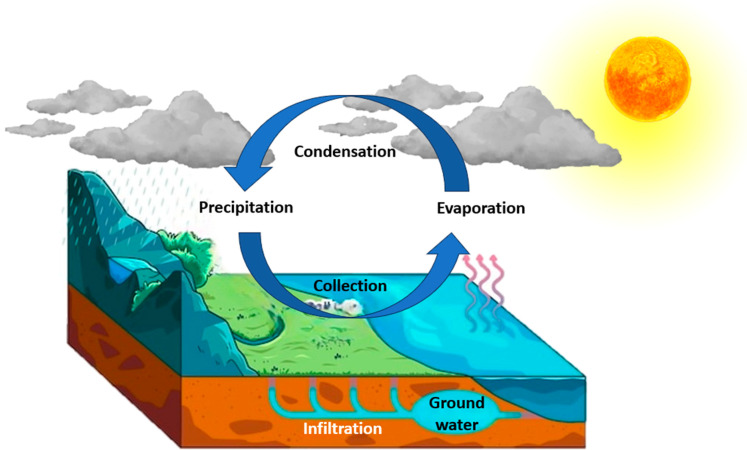
Water hydrologic cycle.

Drinking water primarily comes from groundwater and surface water. Although the Earth is predominantly water, most is saline and unsuitable for human use. Only 0.3% of the Earth’s water is liquid freshwater available for human activities [9]. The greatest proportion of the Earth’s freshwater (98.5%) is present under the earth’s surface, which is known as groundwater or subsurface water, in different geological structures [10]. Groundwater is formed when water precipitates into the ground and collects in the spaces between the rocks and soil particles. In addition, large ground water structures are called “aquifers”. Aquifers are large bodies of porous rocks or sediments that are saturated with groundwater. Aquifers exists all over the world, and they are considered a significant supply source of drinking water [11]. On the other hand, surface water accounts for the remaining small percentage of fresh water and it includes the water that comes from rivers and lakes. It is naturally restored by precipitation and naturally lost by evaporation and discharge to oceans according to the hydrologic cycle (Figure 2).

The amount of water utilized by humans, whether it is groundwater or surface water, varies from one region to another. Generally speaking, groundwater requires less treatment compared to surface water because it is a cleaner source for drinking water. On the contrary, surface water is easier to obtain compared to ground water, which is usually found in aquifers.

## 3. Typical Pollutants Found in Drinking Water

**Figure 3:**
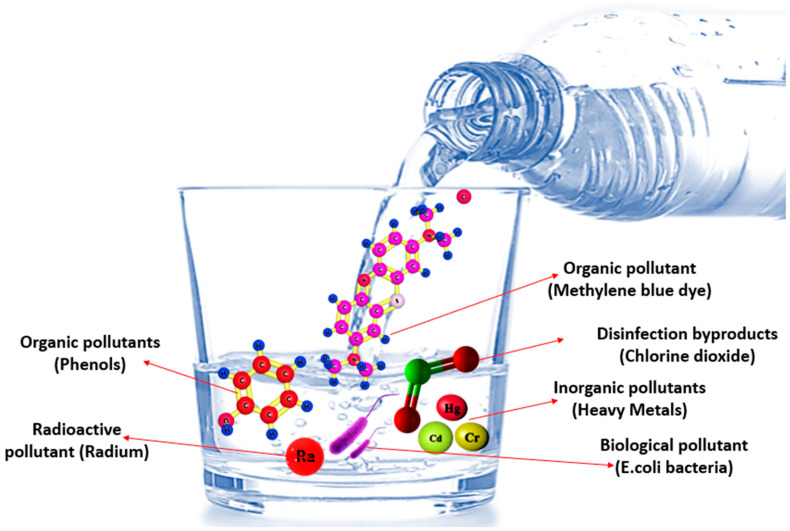
Illustration of the typical types of pollutants found in drinking water.

Generally speaking, drinking water contaminants can be naturally occurring in water or a result of various human activities. Drinking water contaminants are classified by the Environmental Protection Agency (EPA) into the following categories: organic, inorganic, microorganisms, disinfectants, disinfectant by-products, and radionuclides (Figure 3). As a matter of fact, the EPA sets a maximum contaminant level (MCL) for contaminants present in drinking water at which no health side effect can occur upon consumption. The EPA-defined MCLs for common contaminants in drinking water, including organics, inorganics, disinfectants, disinfectant by-products, microorganisms, and radionuclides, are listed in Table 1, Table 2, Table 3, Table 4, Table 5 and Table 6.

Organic contaminants are a group of a human-made chemical compounds that are found in drinking water as a result of their use in a variety of industrial applications, including pesticides, paints, plastics, dyes, and petroleum distillates [12]. Organic contaminants pose serious harm to drinking water health and the aquatic environment as a whole. This is owed to the fact that when they enter the food chain as water pollutants, they affect the taste and odor of fish. Furthermore, large quantities of pesticides and polycyclic aromatic hydrocarbons (PAHs) are introduced into the environment in several agricultural applications. Despite the benefits of pesticides, they also hold several drawbacks including soil and water contamination. In addition, they have several human-health-damaging effects ranging from acute effects (pesticide poisoning) to chronic effects (cancer, neurological damage, reproductive problems, developmental problems, and immune system suppression), which can take decades to cure [13]. Hence, their removal is a must for clean and safe drinking water. Table 1 shows the maximum contaminant levels allowed by the EPA for some common organic pollutants that are found in drinking water along with their potential health side effects in humans.

Inorganic contaminants are compounds that do not contain carbon and include heavy metals (arsenic, cadmium, chromium, copper, lead, mercury, nickel, and zinc), salts, and others. These disastrous contaminates that are usually found in drinking water supplies are human-made chemicals. However, some inorganic contaminants naturally occur in the water of some specific geographic areas. Discharged heavy metals are considered a present threat to human health and natural waters. Heavy metals have a high atomic density and are highly toxic in even small concentrations [14]. Heavy metals enter the water environment through several human activities, including mining, smelting, and manufacturing [15]. Once they enter the environment, heavy metals can remain for extended periods of time and bio accumulate. This means that heavy metals become more concentrated in the tissues of living organisms as they move up the food chain. Even small concentrations of heavy metals in water can lead to high exposure levels for humans and other organisms. Exposure to heavy metals has a variety of adverse health effects on human beings, including cancer, neurological damage, reproductive problems, and developmental delays. Pregnant women and children are more susceptible to these adverse side effects [16]. Furthermore, aquatic habitats can be severely harmed by heavy metals. They have the ability to pollute sediments, destroy food systems, and poison fish and other aquatic life. This results in an overall disruption to aquatic ecosystems and the services they provide to humans. Hence, the removal of heavy metals in drinking water is crucial. Table 2 shows the maximum contaminant levels allowed by the EPA for some common inorganic pollutants that are found in drinking water along with their potential health side effects in humans.

Several types of microorganisms are considered biological contaminants found in water and their removal is a must for a safe drinking water practice. Some microorganisms found in water are harmless, but others can cause severe diseases in humans and animals that ingest them while drinking. Pathogenic microorganisms that contaminate water include the following: bacteria (Escherichia coli, Salmonella, and Vibrio cholera), viruses (hepatitis A virus, rotavirus, and norovirus), protozoa (Giardia lamblia and Cryptosporidium parvum), and helminths (Ascaris lumbricoides and Trichuris trichiura) [17]. The ingestion of water contaminated with pathogenic microorganisms by humans causes sickness with waterborne diseases. In some cases, waterborne diseases can be fatal to human beings. Consequently, their removal from drinking water is crucial. Table 3 show the maximum contaminant levels allowed by the EPA for some common microorganisms that are found in drinking water along with their potential health side effects in humans.

For the sake of killing germs and pathogens from drinking water, some disinfectants are used. The most commonly applied disinfectants in public drinking water systems are chlorine, chloramine, and hydrogen peroxide. These disinfectants are very effective at killing harmful pathogens including bacteria, and other microorganisms that can result in various waterborne diseases. However, when these disinfectants react with organic compounds and molecules that are found in the water, they form disinfection byproducts (DBPs). Some DBPs are linked to severe health problems including cancer and reproductive system problems [18]. Table 4 shows the maximum DBP level goals allowed by the EPA for some common DBPs that are found in drinking water along with their potential health side effects in humans.

Disinfectants can enter drinking water systems from various sources including: improper disposal of disinfectants from wastewater treatment plants, accidental spills or discharges of disinfectants from industrial plants, or agricultural runoff containing disinfectants. Therefore, the removal of disinfectants from drinking water is very important to ensure that no health damage occurs. There is a maximum level of disinfectants allowed set by the EPA for each disinfectant known as the MRDLG (maximum residual disinfectant level goal). Table 5 shows the MRDL allowed set by the EPA for some disinfectants that are commonly used for treating drinking water, along with their potential health side effects.

Radioactive water contaminants (radionuclides) are unstable atoms of certain chemical elements that can emit harmful radiation; these radioactive elements include: cesium, plutonium, and uranium. The emitted radioactive radiation can damage cells and DNA, leading to cancer and other health problems. Radioactive contaminants can enter water drinking systems from several sources including: natural sources, like uranium and radium found in rocks and soil, and humane-made sources, like mining, nuclear power generation, and the production of medical isotopes [19]. Owing to their adverse severe health effects, their removal from drinking water is essential. Table 6 shows the maximum radionuclides levels allowed by the EPA for some common radionuclides that are found in drinking water along with their potential health side effects in humans.

## 4. Overview of the Water Purification Process

**Figure 4:**
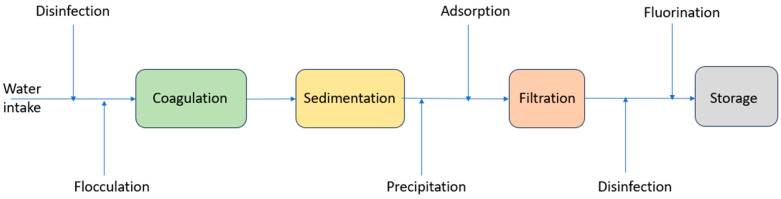
Block flow diagram (BFD) of the water purification process.

Untreated (raw) water passes through a number of treatment steps before reaching our taps. Most of the water treatment techniques applied in the present time were developed at the start of the 20th century. Among these techniques are the traditional methods of treating drinking water, which include flocculation, coagulation, sedimentation, filtration, and chlorine disinfection [20]. Figure 4 shows a block flow diagram of the water purification process. These drinking water treatment methods have several drawbacks that limit their use including: low efficiency, requires long period of time, and high cost. The steps involved in treating and purifying drinking water are as follows: First, chlorine is used as a pretreatment. Chlorine disinfects the drinking water by killing the biological pollutants found in the water, including viruses, bacteria, and protozoa, which can make people unwell and sick. Moreover, chlorine inhibits algae from growing at the treatment plant, which could hamper the water treatment process resulting in taste and odor issues. To make sure that no germs starts to regrow in the distribution system pipelines and residential plumbing, an adequate amount of chlorine is added to the water to retain an ongoing concentration of 1 part per million after filtration [21]. In the second step, the coagulation process takes place. In the coagulation process, the naturally occurring fine physical particulate matter that is suspended in water gets disrupted by adding aluminum sulfate to the water. These contaminants present in water suspensions are made up of particles larger than 0.5 mm and charged colloids with sizes ranging from 5 nm to 1 μm [8]. As a result of the charge and the low density of these particles compared to the water, the majority of these particles will oppose gravity and will not settle in the water. These particles and colloids aggregate when alum is added to the water, forming heavier particles known as flocculocides (floc), which can settle in water. The aggregation of destabilized fine particles into larger masses that are more easily extracted from water is known as flocculation. Third, sedimentation takes place in which the water and floc pass through massive basins called sedimentation or settling basins. Usually, flocculation takes place over a period of 20 to 45 min, while coagulation happens in less than 10 s [22]. During the sedimentation process, floc is eliminated along with other materials like organic matter, and impurities that naturally occur in water. Microbial pollutants, hazardous metals, man-made organic compounds, iron, manganese, and humic materials are some examples of these impurities. Humic-based materials are derived from soil and are created by chemical and biological processes, like vegetation decomposition, in natural water and sediments. Humic based materials should be eliminated from drinking water because they create disinfection by-products during water chlorine treatment [23]. The final stage of water purification involves water filtration by passing the water through a bed of sand and gravel. During the water filtration process, the suspended particles get trapped in the sand bed, and the sand gets separated from the water. Furthermore, fluoride gets added to the filtered water in all water treatment plants to decrease tooth decay. Fluoride levels ranging from 0.7 to 1.2 part per million are maintained in the treated water [24]. Lastly, before the treated water is supplied to the distribution system, lime (calcium oxide) is added. The addition of lime to the treated water causes an increase in its pH level, reaching 8 standard units. This minimizes the water’s tendency to corrode the water mains and the residential plumbing substances including copper, lead, and brass.

Unfortunately, current water purification methods are inefficient and costly, creating a need for better solutions. Nanotechnology offers a cost-effective way to quickly remove impurities, driving research in this area.

## 5. Nanotechnology Application in the Drinking Water Purification Process

Efficient purification processes are vital for safe drinking water, requiring multiple stages to remove pollutants. The choice of technologies depends on the water source and treatment goals, aiming to meet government quality standards. Nanotechnology offers an effective foundation for these processes due to the unique properties of nanomaterials.

Nanomaterials are innovative new materials that are synthesized at a nanoscale level with a size ranging from 1 nm to 100 nm, via a bottom-up technique. Nanomaterials can have properties that are different from the same materials at a larger scale. Applications of nanotechnology have shown great promise in a variety of engineering fields, specifically in the water treatment sector. The application of nanotechnology in the field of water treatment is rapidly expanding, and it has harvested significant attention from researchers, governments, and industries across the globe. This great interest stems from the numerous benefits, properties, and capabilities that nanotechnology offers in addressing the ever-growing challenges related to water quality, availability, and sustainability.

Nanomaterials hold several distinguishable characteristics that makes them remarkable candidates for various applications in the treatment of drinking water. Table 7 shows the unique properties of nanomaterials that helps them in becoming an innovative tool in drinking water purification process.

These unique characteristics allows nanomaterials to be used as an efficient tool in solving the challenges and issues associated with providing safe and clean drinking water. Nanomaterials can be used in several drinking water treatment applications including: pollutant removal, monitoring water quality, improving water infrastructure, and enhancing the efficiency and sustainability water treatment processes. However, to guarantee the safe usage of nanomaterials in drinking water applications, extensive research and risk evaluations should be carried out before choosing the type of nanomaterial [25].

### 5.1. Adsorption Process for Drinking Water Purification

**Figure 5:**
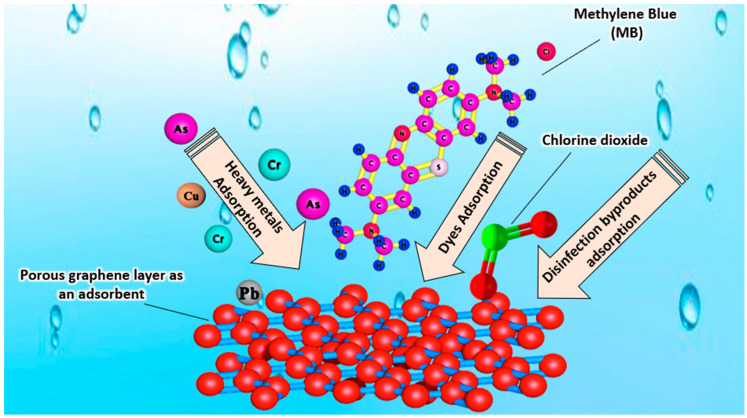
Illustration of the adsorption process of several water contaminants (heavy metals, MB dye, and chlorine dioxide) using porous graphene layer as an adsorbent.

Adsorption is a surface process in which a substance (the adsorbate) in a liquid or gas phase accumulates on the surface of another material (the adsorbent) in a solid or liquid phase (Figure 5). This process is initiated by a physical or chemical attraction between the adsorbate and the adsorbent surface. The adsorption capacity of a substance is affected by several factors including: temperature, impurity type, surface area of the adsorbent, particle size, and contact time. The number and availability of the adsorption sites on the adsorbent surface are mostly determined by the surface area and pore size. As a matter of fact, there is an inversely proportional relationship between surface area and pore size, which means that the smaller the pore size, the larger the surface area. Hence, nanomaterials have a large surface area that ensures a high adsorption capacity owing to their narrow pore size. The conventional adsorbent materials used nowadays for drinking water treatment have low adsorption efficiency owing to their small surface area and low selectivity and adsorption capacity. The most commonly used commercial adsorbent in the present time is activated carbon, which is typically synthesized by heating carbon-rich organic materials at elevated temperatures (>700 °C) [26]. The application of activated carbon as an adsorbent for drinking water treatment is hindered by several factors including regeneration and cost issues. Hence, innovative adsorption materials are required for a more efficient purification process for drinking water.

Nanomaterials are an excellent candidate as an adsorptive material owing to their unique properties, large surface area, abundant sorption sites, tunable pore size and surface chemistry, and ease of regeneration and reuse. Therefore, several studies are focused on the applications of nanomaterials as pollutant adsorbents for the treatment of drinking water.

#### 5.1.1. Carbon-Based Nanomaterial Adsorbents for Drinking Water Purification

One of the most simple and cost-effective methods for removing contaminants from drinking water is adsorption. Carbon-based nanomaterials, including carbon nanotubes, graphene, and its derivatives including graphene oxide, and carbon nanofibers, have great potential application in the purification of contaminated drinking water. Carbon-based nanomaterials show a high adsorption capacity for several types of contaminants in drinking water owing to their large surface area and their functional groups [27].

##### Graphene and Its Derivatives for Drinking Water Purification

**Figure 6:**
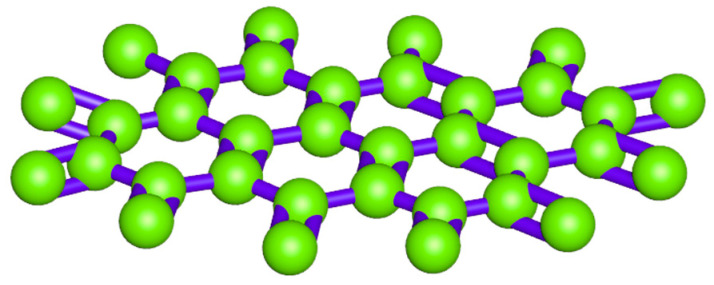
3D model of graphene structure.

As a two-dimensional (2D) carbon material, graphene and its derivatives have received remarkable attention in several research fields owing to their outstanding physicochemical, hydrophobic, and oleophilic properties [28]. Graphene, which has been described by Geim and Novoselov [29] as the ‘mother’ of all graphitic carbon materials, is the building block for graphite and carbon nanotubes (CNTs). Graphene has a hexagonal structure that consists of sp^2^ hybridized carbon atoms (Figure 6). Whitby [30] mentioned that graphene refers to a monolayer of carbon atoms combined into a honeycomb crystal structure, with a one-atomic thickness of sp^2^ bonded carbon that forms a two-dimensional (2D) array of carbon arranged in a hexagonal structure. Aristov, et al. [31] added that the bond length of the carbon–carbon bond in graphene is 1.42 Å. Graphene has enormously attractive optical, thermal, electronic, and mechanical properties. Since the discovery of graphene in 2004, it has been utilized successfully and efficiently in a wide range technologies and industries. Graphene can be synthesized using various methods, including mechanical exfoliation, chemical vapor deposition (CVD), liquid phase exfoliation, and the chemical reduction of graphene oxide (GO). Other techniques include the thermal decomposition of silicon carbide, electrochemical exfoliation, and the pyrolysis of biomass. Each method has a different efficiency, scalability, and cost, making them suitable for specific applications. Tau, et al. [32] mentioned in their study that toluene, o-xylene, and 1,2,3-trimethylbenzene are considered the most favored liquid aromatic precursors for the low-temperature CVD growth of graphene [32]. Paul, et al. [33] synthesized a stacked nanofilm (G-BNG) of pure graphene (G) on boron and nitrogen (BN)-codoped graphene (BNG). The synthesized graphene nano-film had a total thickness of approximately 2–3 nm and 87% transparency at 550 nm. The results of the authors’ study showed that the G-BNG synthesized exhibited a high performance level [33]. One of the most distinguishable properties of graphene is its large surface area, which makes it an excellent adsorbent in the field of drinking water treatment, surpassing other adsorbents in the market [34].

**Figure 7:**
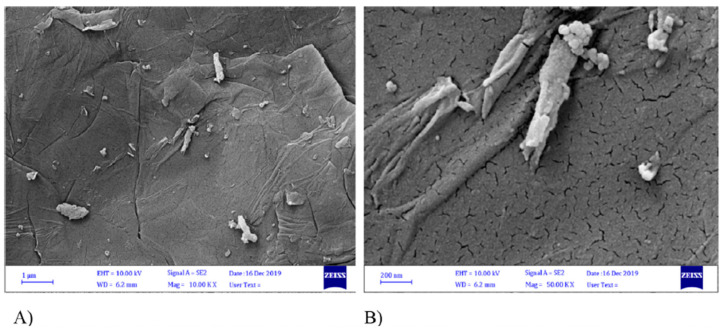
SEM of graphene oxide (GO) at a (**A**) magnification of 10.00 KX and (**B**) magnification of 50.00 KX [35].

The water crisis and rising levels of pollution resulting from anthropogenic sources makes the development of more effective purification materials and technologies for drinking water treatment an urgent concern that needs to be resolved soon. Graphene is studied worldwide both theoretically and experimentally as an adsorbent for water purification technologies. One of the most efficient and remarkable substances that can be applied in the development of technologies and structures for novel adsorbents and drinking water filtration systems is graphene oxide (GO). An SEM image of graphene oxide (GO) at magnifications of 10.00 KX and 50.00 KX is shown in Figure 7 [35].

Kovtun, et al. [36] synthesized two types of GO-doped 3D chitosan-gelatin aerogels (embedded GO aerogels and coated GO aerogels), and investigated their application as an adsorbent for two organic molecules (the fluoroquinolonic antibiotics ofloxacin and ciprofloxacin) and a heavy metal (lead Pb^2+^) from drinking water. The results of the authors’ study show that the maximum adsorption capacity (q_max_) reached for the Pb monolayer was 11.1 mg/g for the embedded GO aerogels, and 1.5 mg/g for the coated GO aerogels. In addition, the use of GO-coated aerogel materials also produced an antimicrobial effects, according to the authors. Therefore, GO aerogels are a remarkable material that can be used efficiently in the removal of contaminants from drinking water, ensuring a highly efficient purification drinking water process [36]. Graphene oxide (GO) is a promising nanomaterial that has demonstrated excellent sorption capacity for pollutants found in drinking water. Masri, et al. [37] studied the ability of graphene oxide as an adsorbent for sulfamethoxazole (SMX) from water. The authors have used several dosages of GO for the removal of SMX and distinct initial concentrations of SMX. The results of the authors’ study show that GO was capable of removing up to 14.40% of SMX from water. Consequently, GO can be effectively applied for the removal of pollutants from water [37]. Parabens have several negative impacts on human health owing to their endocrine disruption activity. Hence, their removal from drinking water is essential to ensure the safe consumption of water. Wei, et al. [38] studied the adsorption and degradation of parabens from water by three types of graphene-family nanomaterials (GFNs) including: graphene oxide, multilayered graphene (MG), and reduced graphene oxide (RGO). The results of the authors’ study show that the adsorption capacities of the given GFNs for parabens followed the following order: reduced graphene oxide (RGO) > multilayered graphene (MG) > graphene oxide (GO). In addition, the paraben adsorption took the following order: butylparaben (BuP) > propylparaben (PrP) > ethylparaben (EtP) > methylparaben (MeP). The results of this study proves that the graphene family (GFN) is highly capable of adsorbing several types of parabens from water to ensure the safe consumption of drinking water [38].

**Figure 8:**
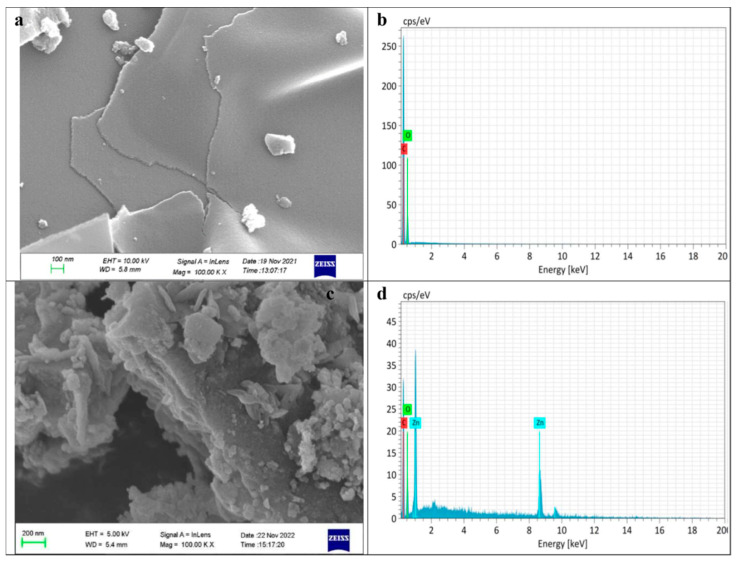
FESEM and EDX of rGO (**a**,**b**) and ZG-40 (**c**,**d**) [41].

In numerous regions of the world, high levels of fluoride (F) in drinking water are a serious issue that affects the well-being of individuals. For the defluoridation of drinking water, economically viable adsorbents are required. Carbon-based nano-adsorbents can be efficiently used for the removal of excess fluoride levels from drinking water. Prathibha, et al. [39] studied the application of graphene oxide-coated sand that is impregnated with zirconium (Zr) for the defluoridation of drinking water. The results of the authors’ study show that the Zr impregnated graphene oxide-coated sand (ZIGCS) had a high fluoride adsorption capacity reaching 6.12 mg/g, which is considered the highest defluoridation value compared to other carbon-based adsorbents reported for defluoridation from drinking water. Therefore, the graphene oxide adsorbent is remarkable in the removal of pollutants from drinking water [39]. Khaliha, et al. [40] investigated the application of graphene oxide (GO) and reduced GO (rGO) nanosheets (GO/rGO) in the removal of methylene blue (MB) from drinking water. The results of the authors’ study show that GO/rGO efficiently removed MB from drinking water with an efficiency three to five times greater than granular activated carbon. Hence, GO/rGO can be efficiently used in the treatment of drinking water [40]. Philip, et al. [41] investigated the use of rGO and rGO/ZnO composite materials in the removal of hexavalent chromium (Cr (VI)) from water. The Cr adsorption studies were carried by the authors using various adsorbents, contact times, initial pHs, and concentrations. The FESEM and EDX of rGO (a and b) and ZG-40 (c and d) are shown in Figure 8.

**Figure 9:**
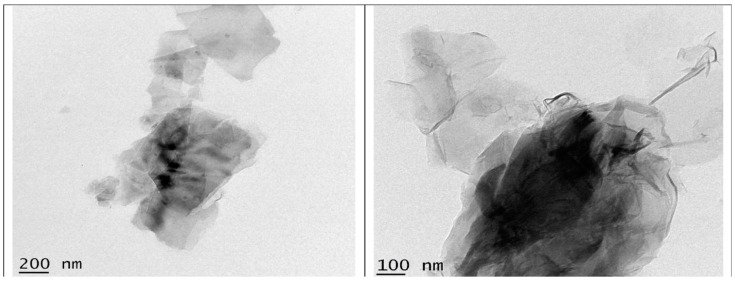
TEM images of rGO at different magnifications [41].

By looking at Figure 8, it is clearly seen that in Figure 8a which shows an FESEM image of rGO, it is illustrated that rGO has a layered, sheet-like structure. Furthermore, the sheets were smooth without any cracks and voids. In addition, the composition of rGO was analyzed by the authors using the EDS spectra, which is shown in Figure 8b. The EDS of rGO shows that rGO is comprised of carbon and oxygen. Furthermore, the absence of any additional peak illustrates that no impurity elements were present in the sample. In addition, the FESEM of the composite sample ZG-40 (the numerical value shows the percentage of zinc acetate used by the authors in the preparation of the corresponding sample) shown in Figure 8c illustrates the layered sheet-like nature for the composite sample ZG-40. Moreover, the EDS analysis of the composite sample ZG-40 shown in Figure 8d shows the presence of the peak corresponding to Zn along with carbon and oxygen. In addition, the authors further confirmed the sheet-like structure of rGO using a TEM image of the sample; see Figure 9.

**Figure 10:**
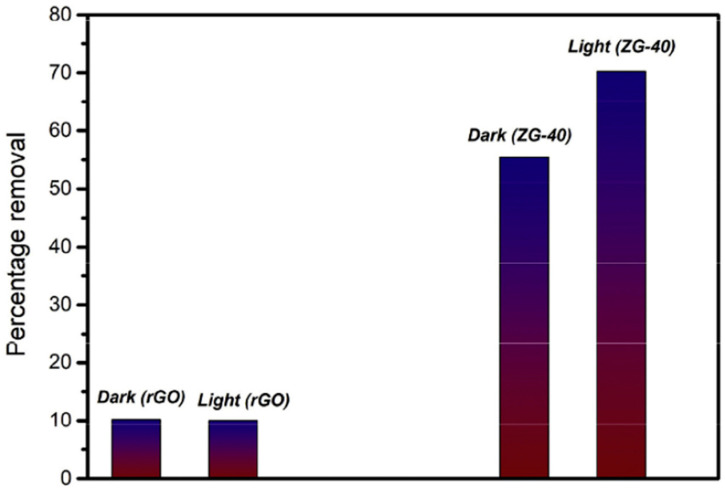
Percentage removal of Cr(VI) by the adsorbents rGO and ZG-40 in dark and light (initial pH—1.5, concentration of the Cr(VI) solution—25 g/L, adsorbent dose—2 g/L, contact time—1 h) [41].

Moreover, the percentage removal of Cr(VI) by rGO and ZG-40 under dark and light was analyzed by the authors’ using a UV–visible spectrophotometer as shown in Figure 10.

**Figure 11:**
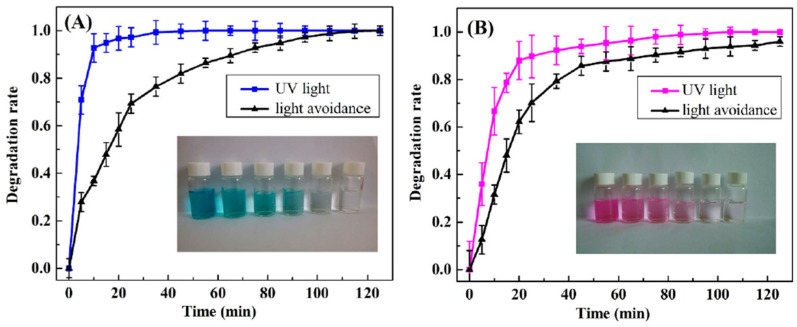
Degradation rate of MB (**A**) and RhB (**B**) solution using rGO/PEI/Ag gel [42].

By looking at Figure 10, it can be concluded that under both dark and light conditions the adsorption efficiency of rGO is the same. This is owed to the reason that rGO does not have any photoactive components. On the contrary, the adsorption efficiency of the rGO/ZnO composite was increased under light irradiation, illustrating the photocatalytic contribution. Furthermore, the percentage removal of Cr(VI) achieved by ZG-40 in the absence of light is 55%, while in the presence of light it is 70%. According to the results of this study, the rGO/ZnO composite is a great candidate that can be used effectively in the removal of Cr(VI) pollutants from water [41]. Jiao, et al. [42] investigated the use of an Ag nanoparticle-containing rGO-based composite hydrogel matrix (rGO/PEI/Ag hydrogels) in the photocatalytic degradation of RhB and MB pollutant dyes from a water solution. The degradation rate of the RhB and MB contaminant dyes is shown in Figure 11.

By looking at Figure 11B, it is shown that the dye degradation rate of RhB reached approximately 100% in almost 70 min. On the other hand, the dye degradation rate of MB was approximately 100% in only 30 min (Figure 11A). According to the results of this study, the as-prepared rGO/PEI/Ag hydrogels were very effective in the removal of MB and RhB dyes from water, and they hold great promise as dye adsorbents for water treatment [42]. Khaliha, et al. [43] investigated the use of defect-rich graphene oxide (dGO) as a sorbent for organic pollutants including drugs and dyes found in drinking water, and the overall performance was compared to graphene types with lower defects. The results of the authors’ study highlights that dGO had a great adsorption capacity for two fluoroquinolone antibiotics (ofloxacin, OFLOX, and ciprofloxacin, CIPRO) that are found in drinking water, with a maximum capacity of 650 mg/g, which is much greater than that for the Hummers-derived commercial GO (hGO) (204 mg/g) and for the less-defected Brodie-derived GO (bGO) for OFLOX (125 mg/g). Furthermore, the highest adsorption capacity of dGO for OFLOX was approximately six times greater than the adsorption capacity of granular activated carbon (95 mg/g) that is used in standard industrial applications. Lastly, it was shown that dGO could be recovered from ultra-filtered treated water, thereby reducing the possibility of secondary contamination and permitting the safe application of graphene nanosheets for drinking water treatment [43]. Microcystin-LR (MC-LR) poses a worldwide environmental threat to the aquatic environment and drinking water supplies. Roberts, et al. [44] investigated the application of graphene in the removal of MC-LR from drinking water supplies. The results of the authors’ investigation shows that the high mesoporosity of graphene resulted in great enhancements to both its kinetics and adsorption capacities toward the removal of MC-LR when compared to microporous granular activated carbon (GAC). Furthermore, graphene exhibited a remarkable MC-LR adsorption capacity reaching 75.4 mg/g, compared to 0.982 mg/g for GAC. Furthermore, employing the same MC-LR concentration, the sorption kinetic experiments revealed that graphene adsorbs 99% of MC-LR in 30 min as opposed to GAC’s zero removal after 24 h. Hence, graphene is an excellent adsorbent for contaminants in drinking water [44]. Tunioli, et al. [45] prepared graphene nanosheets and nanoplatelet–alginate composite hydrogels using ionic gelation, and the gel beads that resulted were investigated for their potential to remove eight selected emerging contaminants (ECs) from drinking water, including bisphenol A, ofloxacin, and diclofenac. The adsorption selectivity, kinetics, and efficiency of the graphene-related materials (GRMs) on the gel beads’ structures were studied by the authors. The results of the authors’ study reveal that GRM had a greater adsorption capacity towards the contaminants compared to the granular activated carbon (GAC), with a 4.3 times greater adsorption capacity of ofloxacin compared to GAC. In addition, a maximum adsorption capacity reaching 178 mg/g for rhodamine B was attained by reduced graphene oxide-based beads. Based on the results of this study, GRMs are superb materials that can be efficiently used for the adsorption of contaminants from drinking water [45].

Lead removal from drinking water is a main topic of interest for several researchers, owing to the great challenge that a trace concentration of lead possesses in drinking water treatment. Shi, et al. [46] prepared a 13X zeolite/reduced graphene oxide composite material (13X-rGO) by using graphene oxide (GO) and 13X zeolite via a chemical reduction mechanism and investigated the composite material usage in the adsorption of trace Pb(II) from drinking water. The results of the authors’ study show that the composite material had a greater adsorption capacity for Pb(II) compared to the single components adsorption. In addition, within the range of 30 s the effluent lead concentration could be lowered to the allowed limit permitted by the World Health Organization (WHO) (<10 ppb). Furthermore, the prepared 13X-rGO showed a superb adsorption capacity of 6.07 mg/g for lead, which meets the WHO standards, and is approximately 2.23 times greater than the adsorption capacity of single 13X zeolite (2.72 mg/g). According to the results of this study, 13X-rGO can be efficiently used as an adsorbent for lead from drinking water, ensuring a highly efficient drinking water purification process that meets the WHO standards and regulations for drinking water [46]. Chlorine oxyanions including hypochlorite ClO^−^, chlorite ClO_2_^−^, chlorate ClO_3_^−^, and perchlorate ClO_4_^−^ are disinfectant by-products and are typically present in the disinfection process of drinking water treatment plants (DWTPs). The presence of chlorine oxyanions in drinking water is considered harmful to human health and their removal is crucial to ensure the safe consumption of drinking water. Elgengehi, et al. [47] studied the adsorption of chlorine oxyanions on graphene flakes (Gr) from drinking water. The results of the authors’ study show that graphene flakes were efficient in the removal of chlorine oxyanions from drinking water, and can be successfully used in drinking water purification processes [47]. Majdoub, et al. [48] investigated the adsorption capacity of graphene oxide (GO) towards heavy metals, including Cd (II), Cu (II), and Pb (II), that are found in drinking water. The results of the authors’ study show that the adsorption rates attained were 100% for Pb (II), 98.18% for Cu (II), and 95.19% for Cd (II). These high adsorption rates of heavy metals prove that graphene oxide is a superb adsorbent material that can be efficiently used in the purification process of drinking water [48]. Karabörk, et al. [49] investigated the application of graphene oxide (GO) as an adsorbent material of cobalt (II) and zinc (II) ions from drinking water. The results of the authors’ study show that graphene oxide had a high adsorption rate for cobalt (II) and zinc (II) ions from drinking water. Hence, graphene oxide can be successfully used as an adsorbent material for heavy metal drinking water purification technologies [49]. The carcinogenic effect of hypertoxic Cr(VI) on the human body necessitates its removal from drinking water to ensure safe water consumption. Sun, et al. [50] synthesized a graphene oxide-based nanoadsorbent co-functionalized with polydopamine and branched polyethyleneimine (GOPP) for the removal of Cr(VI) from water. The results of the authors’ study demonstrated that GOPP was capable of removing 85.8% of Cr(VI) from water. Hence, graphene-based adsorbents can be successfully used in drinking water purification processes [50]. Zhang, et al. [51] synthesized an active Fe(III) oxyhydroxide-Mn(IV) oxide/3D graphene oxide (GO) gel composite using a simple hydrothermal reaction for the removal of arsenic As(III, V) from drinking water. The results of the authors’ study show that the fabricated Fe–Mn/GO composite showed great adsorption performance with a removal efficiency of greater than 90% for As(III) at pH 7.0, and approximately 97% for As(V) at pH 5. Hence, Fe–Mn/GO composites are promising candidates in the purification process of drinking water for contaminant removal [51]. Lebron, et al. [52] fabricated GO sheets for heavy metal adsorption from drinking water. The results of the authors’ study demonstrated that GO successfully removed more than 90.2% of the copper, manganese, and aluminum metals found in the drinking water. Therefore, GO is a superb adsorbent for heavy metals from drinking water and can be applied efficiently in drinking water treatment applications [52]. Yang and Cao [53] synthesized graphene oxide (GO) via a modified Hummers method, and under strong alkali conditions carboxylated graphene oxide (GO-COOH) was further fabricated by the derivatization reaction of bromoacetic acid with a hydroxyl group. The resultant GO and GO-COOH were studied for their usage as adsorbents for copper and lead ions from drinking water. The results of the authors’ study show that the maximum adsorption capacity of GO was increased after treatment with the carboxyl group from 55.47 mg/g to 62.02 mg/g for Cu(II), and from 85.17 mg/g to 96.21 mg/g for Pb(II). Thus, GO and GO-COOH are a great adsorbents for Cu(II) and Pb(II) from drinking water, and can be efficiently utilized in drinking water purification processes [53].

Anthropogenic activities have a catastrophic impact on water contamination and freshwater availability. The uncontrolled usage of hazardous chemicals in the environment and industrial plants results in water resource pollution, and, consequently, leads to severe health issues. The application of graphene-based materials in the adsorption process has been investigated as a cost-friendly technique for heavy metal elimination during drinking water treatment processes. Kumar, et al. [54] investigated the use of magnetic graphene-based composites in the adsorption of various heavy metals, including arsenic (III)/(V), chromium (III)/(VI), lead (II), and selenium (IV)/(VI), that are found in drinking water. The results of the authors’ study reveal that magnetic graphene-based composites efficiently adsorbed all of the specified heavy metals from drinking water. Therefore, magnetic graphene-based composites are superb candidates that can be used efficiently for the removal of heavy metals during drinking water treatment processes [54]. Bloor, et al. [55] investigated the usage of aerogel fabricated from graphene oxide (GO) cross-linked with alginate for the removal of Pb^2+^ from drinking water. The results of the authors’ study show that the maximum adsorption capacity attained by the GO alginate aerogel was 504 mg/g of Pb^2+^ in 240 min at pH 5. In addition, the aerogel exhibited adsorption for other toxic metal salts including La^3+^ and Cu^2+^ with adsorption capacities of 146 and 193 mg/g, respectively. Based on the results of this study, GO alginate aerogels are highly efficient in the removal of Pb^2+^ from drinking water and can be successfully applied in drinking water purification processes [55]. Al-Ghouti, et al. [56] synthesized graphene oxide (GO) nanoparticles using two distinct techniques, through a hybrid adsorption–UV-irradiation process, and investigated its usage in the removal of phenol from drinking water at several pH levels. The results of the authors’ study showed that the UV-irradiation synthesized GO had a higher adsorption capacity towards phenols compared to the GO that was not exposed to UV irradiation. In addition, the maximum adsorption capacity towards phenols was increased with the effect of UV exposure from 70.43% to 90.82%. This study reveals that GO adsorbents are highly efficient in the removal of phenols from drinking water [56]. Deb, et al. [57] studied the application of porous graphene nanomaterials (PGNs) in the removal of methyl blue (MB) from drinking water. The results of the authors’ study show that the PGNs demonstrated a high adsorption capacity towards MB, reaching 821.05 mgg^−1^ (99.69%) within 120 min. Thus, graphene-based nanomaterials are very promising adsorbents for contaminants in drinking water [57]. Shahzadi, et al. [58] synthesized a cellulose graphene material sandwiched between a double-layered polydopamine (PDM) metal–organic framework (MOF), and investigated its usage in the removal of heavy metals (Pb^2+^ and Hg^2+^) from drinking water. The results of the authors’ study showed that the composite material successfully eliminated the two water pollutants, Pb^2+^ and Hg^2+^. The results of this study show that graphene-based materials can be efficiently used in the removal of heavy metals from drinking water [58]. Table 8 shows a summary of graphene-based adsorbents in water purification applications.

##### Carbon Nanotube (CNT)-Based Adsorbents for Drinking Water Purification

**Figure 12:**
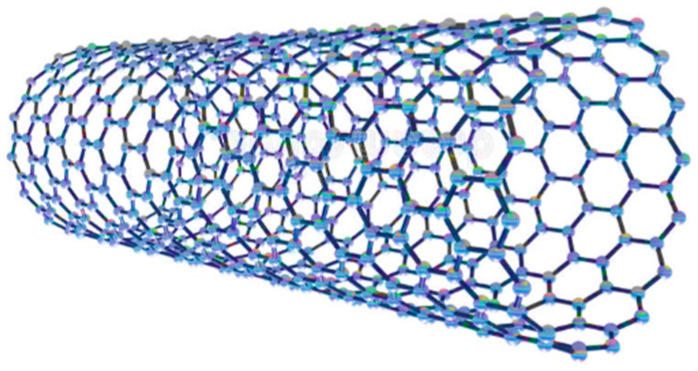
Carbon nanotube (CNT) 3D structural model.

Carbon nanotubes (CNTs) are cylindrical nanostructures that consists of carbon atoms. CNTs are very light in weight and have a strong rigid structure. The versatility of CNTs allows their application in several industries and applications, including water treatment, electronics, optics, and medicine. For example, buckypaper, which is a material derived from a network of CNTs, is capable of retaining the exceptional properties of CNTs, making it lightweight, strong, and flexible. Buckypaper functions as an effective electrode in batteries and supercapacitors. Basiuk, et al. [97] investigated the solvent-free functionalization of buckypaper (BP) mats prefabricated from oxidized multi-walled carbon nanotubes (MWCNTs-ox). The results of the authors’ study showed that the synthesized buckypapers had an exceptional water stability and high electrical conductivity [97]. CNTs are formed when a sheet of carbon atoms is rolled up into a cylindrical shape (Figure 12). The shape and size of the CNT varies according to the carbon atoms’ arrangement. CNTs can be categorized as follows: single-walled or multi-walled CNTs. Single-walled CNTs (SWCNTs) consist of a single sheet of carbon atoms, while multi-walled CNTs (MWCNTs) consist of multiple layers of carbon atoms. CNTs have several remarkable properties that distinguish them from other materials, including very high tensile strength, high stiffness, great electrical conductivity, and high thermal stability and conductivity. Owing to these superb properties, the application of CNTs as an adsorbent for contaminants in drinking water treatments processes has been widely investigated.

**Figure 13:**
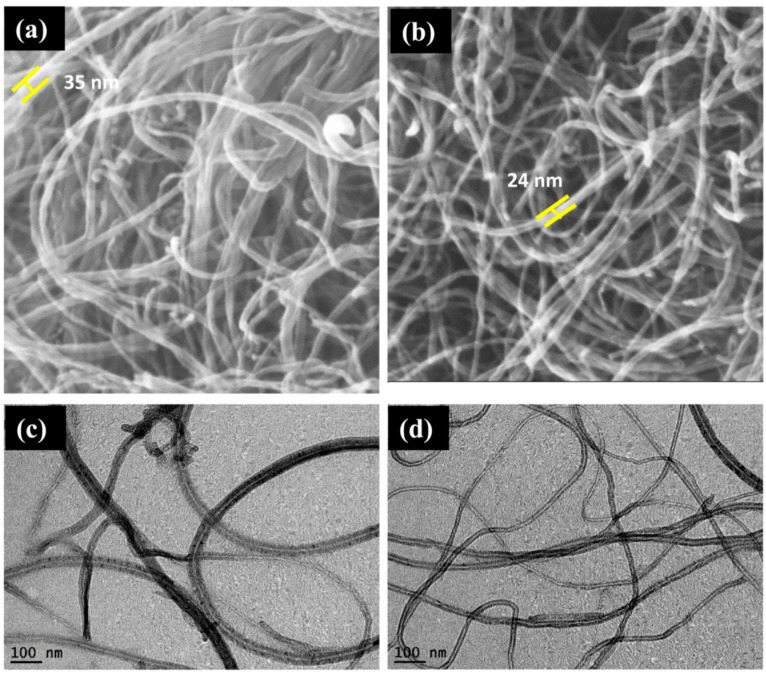
SEM images of MWCNT: (**a**) raw and (**b**) functionalized CNTs. TEM images for raw tubes (**c**) and functionalized CNTs (**d**) [98].

Deepanraj, et al. [98] investigated the use of functionalized multi-walled carbon nanotubes (F-MWCNTs) for the removal of methylene blue (MB) dye from water. SEM and TEM images of the MWCNTs before and after functionalization are shown in Figure 13.

**Figure 14:**
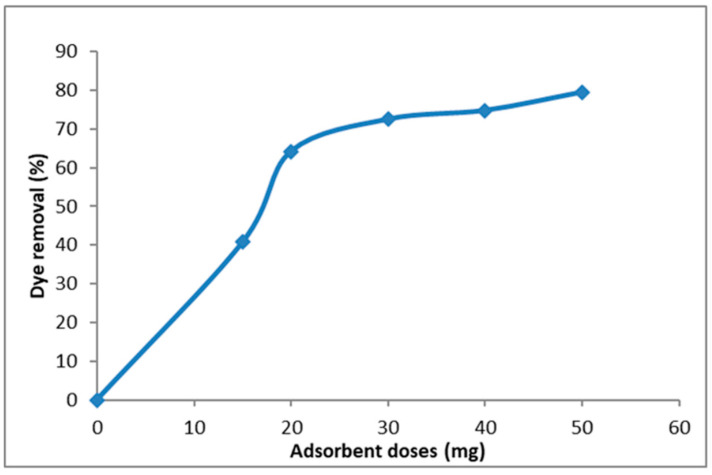
The influence of the adsorbent F-MWCNTs’ mass on the MB removal efficiency [98].

It can be concluded from Figure 13 that the length and diameter of the raw CNTs is greater than that of the functionalized CNTs tubes. Furthermore, the maximum removal percentage of MB dye obtained by F-MWCNTs is 80% as can be seen in Figure 14.

**Figure 15:**
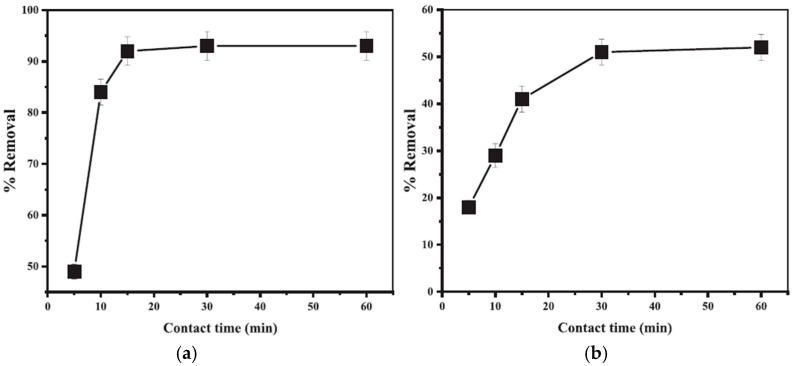
Percentage removal of CV (%) versus contact time (min): (**a**) CoFe-PB/CNT and (**b**) CNT. (CV concentration 5 mg/L, adsorbent dosage = 50 mg, pH = 6) [99].

By looking at Figure 14, it is clear that the maximum MB dye percentage removal reached was 80% using 50 mg of F-MWCNTs. Based on the results of this study, functionalized MWCNTs hold great promise in the removal of MB dye from water and can be applied in water remediation systems [98]. Alanazi [99] investigated the use of CNT/cobalt hexacyanoferrate core/shell nanostructure (CoFe-PB/CNT) for the removal of crystal violet (CV) dye from water. The CV concentration was measured by the authors via a spectrophotometer. The relationship between the CV dye removal efficiency and the contact time with the adsorbent is shown in Figure 15.

**Figure 16:**
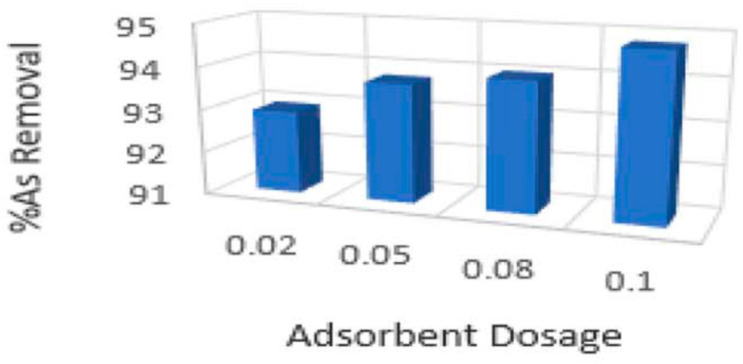
Effect of adsorbent dosage on arsenic removal efficiency (pH = 2, arsenic concentration = 6 mg L^−1^) [100].

By looking at Figure 15, it is clear that the initial adsorption rate is faster on the surface of the CoFe-PB/CNT adsorbent and then slows down owing to the internal diffusion phenomena. After 15 min, a constant percentage removal can be clearly seen. Therefore, the time required to reach equilibrium was 15 min for CV dye using CoFe-PB/CNT. In addition, the CV percentage removal using CNT had a slower rate compared to CoFe-PB-CNT. In addition, the time required to reach equilibrium was 30 min for CV using CNT. Thus, CoFe-PB/CNT had faster kinetics compared to CNT. According to the results of this study, CNT modified with CoFe-PB is a remarkable candidate for the remediation of CV dye from water [99]. Khorasani Alamdari, et al. [100] studied the use of magnetized multi-walled carbon nanotubes with iron oxide (Fe_3_O_4_) nanoparticles in the removal of arsenic (As) from water. The effect of the adsorbent dosage on the percentage removal of arsenic is shown in Figure 16.

By looking at Figure 16, which shows the effect of adsorbent dosage on arsenic removal efficiency, it is clear that the maximum As removal % reached was approximately 94.5% at an adsorbent dosage of 0.1 g L^−1^. According to the results of this study, the proposed magnetized multi-walled carbon nanotubes with iron oxide (Fe_3_O_4_) nanoparticles can be successfully applied in the remediation of water from arsenic contaminants [100].

Diazinon is an organophosphate insecticide that is widely used in agriculture, and for household pest control. Diazinon is highly toxic to human health; hence, the removal of any traces of diazinon from drinking water is crucial. Fu, et al. [101] investigated the use of single-walled carbon nanotubes (SWNTs) and multi-walled carbon nanotubes (MWNTs) in the adsorption of diazinon from drinking water. The results of the authors’ study show that five-walled CNTs resulted in the greatest adsorption capacity of diazinon from drinking water, owing to their high interactions with diazinon. Hence, CNTs can be efficiently applied as an adsorbent for pesticides from drinking water [101]. In the present time, the excessive usage of agricultural fertilizers for the purpose of industrial development has resulted in an increased production level of poisonous inorganic ions including nitrates in water and soil. The presence of nitrates in drinking water is a potential health risk that causes severe problems for humans. The elimination of nitrates from drinking water is a great challenge for researchers. Altowayti, et al. [102] investigated the application of multi-wall carbon nanotubes functionalized with mesoporous silica-nitrenium ions (CNT-MS-N) as a novel adsorbent for the elimination of nitrate ions (NO_3_^−^) from drinking water. The results of the authors’ study show that the maximum removal efficiency of nitrate ions (NO_3_^−^) by CNT-MS-N reached 98%. The results of the present study confirmed the ability of carbon nanotubes to adsorb nitrates from drinking water [102]. Mahpishanian, et al. [103] studied the usage of porous nanocomposites containing single-walled carbon nanotubes (SWCNTs) for the removal of Cr(VI) from drinking water. The results of the authors’ study show that the maximum adsorption capacity reached by the nanocomposite containing SWCNT for Cr(VI) was 72.35 mg g^−1^. This high adsorption capacity is owed to the great porosity of the nanocomposite. Therefore, CNT-based adsorbents are excellent candidates for the removal of heavy metal ions from drinking water [103]. Wang, et al. [104] synthesized ionic liquid@β-cyclodextrin-reduced graphene oxide-multi-wall carbon nanotubes (IL@β-CD-rGO-MWCNTs) as an adsorbent for bentazon from water. The results of the authors’ study demonstrated that the maximum adsorption capacity reached by IL@β-CD-rGO-MWCNTs for bentazon was 125.00 mg g^−1^. Hence, CNTs based adsorbents can be successfully applied in drinking water purification technologies for the removal of contaminants [104].

Phenolic compounds are typical industrial contaminants that possess huge negative impacts on the water environment and human health. Hence, the fabrication of efficient adsorbents for phenolic compounds from drinking water is extremely important. Gao, et al. [105] fabricated HCNTs/Fe_3_O_4_ composites via a co-precipitation method by loading magnetic Fe_3_O_4_ particles onto hydroxylated multi-walled carbon nanotubes (MWCNTs), and investigated their usage as an adsorbent for bisphenol A (BPA) and *p*-chlorophenol (*p*-CP) from drinking water. The results of the authors’ study show that HCNTs/Fe_3_O_4_ had maximum adsorption capacities of 41.6 mg g^−1^ for *p*-CP and 113 mg g^−1^ for BPA at 303 K. In addition, the removal efficiencies were 88% for BPA and 66% for *p*-CP. Based on the findings of this study, the HCNTs/Fe_3_O_4_ composite is an efficient adsorbent for BPA and *p*-CP from drinking water. Hence, it can be used efficiently for the removal of contaminants from drinking water [105]. Addo Ntim and Mitra [106] investigated the use of a multi-wall carbon nanotube–zirconia nanohybrid (MWCNT–ZrO_2_) as an adsorbent for arsenic from drinking water. The results of the authors’ study show that MWCNT–ZrO_2_ with 4.85% zirconia was successful in achieving the drinking water standard levels of 10 μg L^−1^. Furthermore, the adsorption capacity of MWCNT–ZrO_2_ for As(III) was 2000 μg g^−1^ and 5000 μg g^−1^ for As(V). These high adsorption capacities of arsenic proves that CNT-based adsorbents are highly efficient in the removal of heavy metals from drinking water and can be effectively applied in drinking water purification technologies [106]. Marszałek, et al. [107] fabricated bentonite–carbon nanotubes composites (Ben–CNT) for the adsorption of organic micropollutants and heavy metals, including lead nitrate, zinc nitrate, benzotriazole (BZT), and anthracene (ANT), from drinking water. The results of the authors’ study show that the removal of BZT reached 93%, and the removal of ANT was up to 95.5%. In addition, the composite demonstrated a 90% removal of zinc and lead. Consequently, Ben–CNT composites are effective adsorbents for the elimination of micro contaminants and heavy metals from drinking water [107]. Abutaleb, et al. [108] investigated the application of an Fe_3_O_4_-multi-walled carbon nanotube (MWCNT)–bentonite nanocomposite as an adsorbent for the removal of methylene blue (MB) dye from drinking water. The results of the authors’ study showed that the Fe_3_O_4_-multi-walled carbon nanotube (MWCNT)–bentonite nanocomposite CNT-based nanocomposite exhibited an adsorption capacity of 48.2 mg/g for MB dye. This result proves that CNT-based adsorbents are capable of efficiently adsorbing dyes from drinking water [108]. Bakather [109] studied the usage of multi-walled carbon nanotubes impregnated with Fe_2_O_3_ nanoparticles for the removal of benzene from drinking water. The results of the study showed that the maximum adsorption capacity of impregnated CNTs was found to be 271 mg/g for benzene from drinking water. As a result, CNT-based adsorbents are outstanding materials for the removal of benzene from drinking water [109]. Saleh, et al. [110] synthesized carbon nanotubes functionalized with amine groups and conjugated with two low-symmetry prophyrine derivatives, and investigated their application as an adsorbent of methylene blue (MB) from water. The results of the authors’ study show that the maximum adsorption efficiency of MB reached was approximately 100%. Based on this result, CNT-based adsorbents are an excellent material that can be applied efficiently for the removal of dyes from drinking water [110].

Copper (Cu(II)) water pollution is very toxic to human health, and designing adsorbents that are capable of removing copper from drinking water is very challenging. Zhao, et al. [111] synthesized novel adsorbent MnFe_2_O_4_/multi-wall carbon nanotubes (MMWCNTs) for the removal of copper from drinking water. The results of the authors’ study show that the maximum adsorption capacity reached by MMWCNTs for Cu(II) was 46.41 mg/g at 308 K. These results reveal that MMWCNTs are an excellent adsorbent that can be efficiently applied in the removal of contaminants from drinking water to ensure safe water consumption [111]. Al-Musawi, et al. [112] fabricated multi-walled carbon nanotubes coated with CoFe_2_O_4_ nanoparticles for the adsorption of bisphenol A (BPA) from drinking water. The maximum adsorption capacity reached by MWCNTs/CoFe_2_O_4_ for BPA removal was 416.6 mg/g, which is greater than that of other reported adsorbents applied for the same purpose. This study revealed that MWCNTs/CoFe_2_O_4_ are an efficient adsorbent for BPA with a high removal efficiency reaching 99%. Hence, it can be efficiently used for the remediation of drinking water from contaminants [112]. Sarkar and Paul [113] investigated the use of zirconia-multi-walled carbon nanotube nanoheterostructures in the adsorption of As(V) from drinking water. The results of the authors’ study showed that the zirconia-multi-walled carbon nanotube efficiently adsorbed As(V) from drinking water. Hence, it can be efficiently used as an adsorbent for heavy metals from drinking water [113]. Pereira, et al. [114] fabricated two novel materials with functionalized multi-walled carbon nanotubes (MWCNT–OH and MWCNT–COOH) that are impregnated with magnetite (Fe_3_O_4_) via a solution precipitation methodology. The resulting MWCNT–OH–Mag and MWCNT–COOH–Mag materials were tested for the adsorption of the pollutants 2,4-D and atrazine from drinking water. The results of the authors’ study show that the greatest adsorption capacities of the specified contaminants were reached by MWCNT–OH–Mag with 51.4 and 47.7 mg g^−1^ adsorption capacities for 2,4-D and atrazine, respectively. Furthermore, MWCNT–COOH–Mag showed a superior removal efficiency for both contaminants. Hence, CNT-based adsorbents can be efficiently used in the removal of contaminants from drinking water [114]. Cheng, et al. [115] investigated the use of a new nanoscale iron oxide (FeO_x_)-modified carbon nanotube composite (FeO_x_@CNT) in the removal of Sb(III) from drinking water. The results of the authors’ study show that the maximum adsorption capacity attained by the FeO_x_@CNT for Sb(III) was 172 mg/g. Based on the results of this study, the FeO_x_@CNT nanocomposite is capable of efficiently adsorbing Sb(III) from drinking water to provide clean drinking water for consumers [115]. Gupta, et al. [116] investigated the application of functionalized multi-walled carbon nanotubes (F-MWCNTs) as an adsorbent for the removal of Cu (II) ions from drinking water. The results of the authors’ study show that the maximum adsorption capacity of Cu (II) ions reached was 118.41 mg g^−1^, with a removal efficiency of approximately 93% from water. Consequently, the adsorption of Cu (II) ions from drinking water using F-MWCNTs as an adsorbent is a successful and efficient process [116]. Ma, et al. [117] fabricated an iron oxide nanoparticle-coated SWCNT composite, and investigated its application as an adsorbent for As^5+^ removal from drinking water. The results of the authors’ study show that the maximum adsorption capacity reached for As^5+^ removal was 49.65 mg g^−1^. Therefore, SWCNT composite-based adsorbents are remarkable candidates that can be used efficiently in the removal of heavy metal ions from drinking water [117]. Egbosiuba, et al. [118] studied the application of MWCNTs and carboxylated MWCNTs as adsorbents for As(V) and Mn(VII) from drinking water. The results of the authors’ study demonstrated that the adsorption capacity of As(V) and Mn(VII) increased from 192 mg/g for MWCNTs to 298 mg/g for MWCNTs-OCH_2_CO_2_H. Hence, MWCNTs and MWCNTs-OCH_2_CO_2_H are highly promising candidates that can be used as adsorbents for the removal of heavy metal ions from drinking water [118].

Atieh [119] studied the use of carbon nanotubes supported by activated carbon as an adsorbent for the removal of chromium (VI) ions from polluted drinking water. The results of the study show that activated carbon coated with carbon nanotubes is a superb adsorbent for Cr (VI) ions from drinking water with an adsorption capacity of 9.0 mg/g [119]. Ansari, et al. [120] studied the application of multi-walled carbon nanotubes (MWCNTs) as an adsorbent for the elimination of fluoride (F^−^) from drinking water. The results of the study revealed that the maximum adsorption capacity reach by MWCNTs for F^−^ was 3.5 mg/g with an adsorption efficiency of approximately 85% of the total fluoride content found in drinking water. Thus, MWCNTs is a remarkable candidate that can be used as an adsorbent for drinking water purification processes to remove contaminants [120]. Dehghani, et al. [121] investigated the application of multi-walled carbon nanotubes (MWCNTs) as an adsorbent of organophosphorus pesticide malathion from drinking water. The results of the authors’ study showed that the maximum adsorption capacity of malathion reached was 6 mg/L. Hence, MWCNTs can be efficiently applied as an adsorbent for the removal of pesticides from drinking water to ensure its safe consumption [121]. Table 9 shows a summary of carbon nanotube (CNT)-based adsorbents in water purification applications.

### 5.2. Application of Engineered Nanomaterials in Drinking Water Purification

#### 5.2.1. Applications of TIO_2_ Nanoparticles in Drinking Water Purification

**Figure 17:**
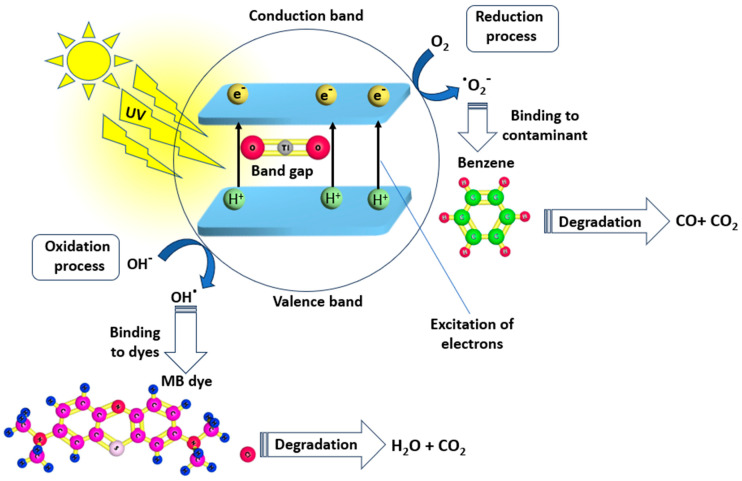
The photocatalytic activity of TiO_2_ nanoparticles under UV irradiation. The exposure of TiO_2_ nanoparticles to UV irradiation results in the formation of radicals that are capable of binding to organic and inorganic contaminants, leading to their degradation.

**Figure 18:**
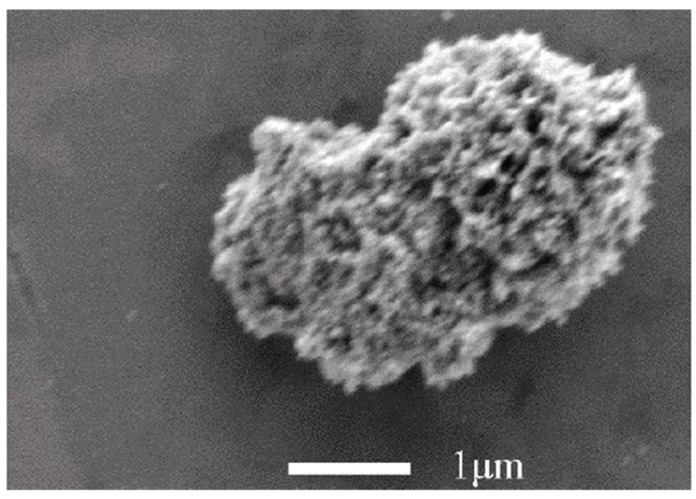
SEM image of pure TiO_2_ [143].

As can be seen from the SEM image of pure TiO_2_ in Figure 18, TiO_2_ has a loose block-like structure with a large volume [143]. Under ultraviolet (UV) light exposure, photocatalysis occurs, where TiO_2_ NPs produce highly reactive free radicals that are capable of breaking down organic pollutants, microorganisms, and inorganic contaminants in drinking water. Figure 17 shows the photocatalytic activity of TiO_2_ nanoparticles when exposed to UV irradiation. Several studies have focused on the antimicrobial activity of TiO_2_ nanoparticles against several waterborne pathogens including bacteria (*Salmonella*, *Mycobacteria*, and *Shigella*) and protozoa (*Giardia duodenalis* and *Entamoeba histolytica*). According to research, the ions of TiO_2_ released by TiO_2_ nanoparticles trigger a variety of biochemical and physical changes in microbial cells, which ultimately cause the cells to die. The high photocatalytic activity is the most crucial and unique property of TiO_2_ nanomaterials. Generally speaking, photocatalysis is defined as a chemical process in which a substance’s activity is initiated by the absorption of photons to accelerate a specific reaction. Photocatalysis includes two steps: In the first step, energy is transferred to the substrate in the form of photons, which activates the substrate. In the second step, oxidation occurs, which is made easier by the substrate’s activated state. In addition, there are three distinct types of TiO_2_ crystals: rutile, brookite, and anatase. The most photocatalytically active type of crystal amongst those different forms is anatase. The following steps describe the mechanism of the photocatalytic activity of TiO_2_; the valence electron in TiO_2_ becomes excited when exposed to a photon with enough energy, moving from the valence band to the conduction band and creating a hole in the valence band. The oxidizing behavior of TiO_2_ makes it a superb material for the degradation of inorganic and organic materials.

Titanium nanoparticles (TiO_2_ NPs) have emerged as a promising material for drinking water purification technologies owing to their unique photocatalytic properties, remarkable antimicrobial activity, and their non-toxic nature. Figure 18 shows an SEM image of pure TiO_2_ [143].

**Figure 19:**
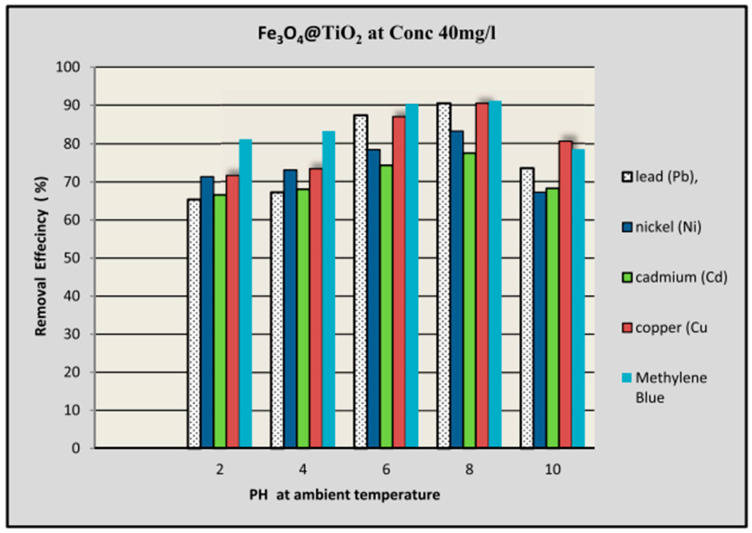
Removal efficiency (%) of heavy metals (Cu, Ni, Pb, and Cd) and methylene blue dye (MB) from water using Fe_3_O_4_@TiO_2_ at 40 mg/L [144].

Owing to these remarkable properties of TiO_2_ nanoparticles, their application in water purification technologies has been widely investigated. Almutairi [144] investigated the use of the metal oxide Fe_3_O_4_@TiO_2_ in the removal of heavy metals (Cu, Ni, Pb, and Cd) and methylene blue dye (MB) from wastewater. The authors investigated the removal efficiencies (%) of the contaminants using Fe_3_O_4_@TiO_2_ at various pH levels; see Figure 19.

By looking at Figure 19, it is clearly seen that the highest removal efficiencies of all contaminants were achieved at pH 8 and 40 mg/L of Fe_3_O_4_@TiO_2_. The highest removal efficiencies of the contaminants at pH 8 are as follows: MB (91.16%) > Pb (90.5%) > Cu (90.48%) > Ni (83.34%) > Cd (77.58%). This study demonstrates that Fe_3_O_4_@TiO_2_ is a promising material for water treatment, offering cleaner and safer water for better human health and a better environment [144].

Surendra, et al. [145] investigated the use of TiO_2_ nanoparticles in the removal of a herbicide known as atrazine from water. The results of the authors’ study show that TiO_2_ nanoparticles were capable of removing 76.15% of the atrazine. This result shows that TiO_2_ nanoparticles are potential candidates for the removal of contaminants from drinking water [145]. Phattepur, et al. [146] fabricated zinc-doped black TiO_2_ nanoparticles via a modified sol–gel method, and investigated its usage in the degradation of 2, 4, 6 tri-chloro-phenol from water. The results of the authors’ study demonstrated that zinc-doped black TiO_2_ nanoparticles were capable of degrading up to 95% of the 2, 4, 6 tri-chloro-phenol found in water. Hence, TiO_2_ nanoparticles are capable of removing contaminants from water [146]. Singh, et al. [147] investigated the application of TiO_2_ and ZnO nanoparticles in the removal of microbes from drinking water. The results of the authors’ study show that, under ultraviolet (UV) irradiation, ZnO and TiO_2_ demonstrated superb potential as UV-absorbing and photocatalytic materials, and can be used in the removal of microbes from drinking water [147]. Kaur, et al. [148] studied the use of an *E. cardamomum*-derived TiO_2_ photocatalyst in the degradation of Congo red dye from water. The results of the authors’ study revealed that the *EC*:TiO_2_ exhibited a high photocatalytic degradation efficiency up to 97% towards CR dye. In addition, *EC*:TiO_2_ NPs have shown great antibacterial effect against two bacterial species (*S. aureus* and *P. aeruginosa*) that are found in water. According to the results of this study, *EC*:TiO_2_ NPs are a promising photocatalyst that can be applied efficiently in the removal of dyes and bacterial pathogens from drinking water to ensure safe water consumption [148].

The emergence of several types of contaminants in drinking water is of great concerns nowadays. Specifically, pharmaceutical contaminants which exhibit a high persistency in water owing to their difficulty in removal using conventional treatment technologies cause detrimental environmental issues. Hence, the emergence of new technologies and materials for the removal of these persistent contaminants is a must. Zheng, et al. [149] investigated the use of TiO_2_- and ZnO-nanoparticle-based photocatalysts in the photocatalytic degradation of four highly abundant pharmaceuticals in water: paracetamol (PAR), chloroquine phosphate (CLQ), diclofenac sodium (DCF), and ciprofloxacin (CIP). The results of the authors’ study demonstrated that TiO_2_- and ZnO-nanoparticle-based photocatalysts had high degradation efficiencies for all the specified pharmaceuticals. Hence, TiO_2_-nanoparticle-based photocatalysts are a promising material that can be efficiently used in the removal of contaminants from drinking water [149]. Gora and Andrews [150] investigated the usage of titanium dioxide NPs as a photocatalyst for the elimination of natural organic matter (NOM) and disinfection byproduct (DBP) precursors from drinking water, under UV light irradiation. The results of the authors’ study show that the TiO_2_ nanoparticles exhibited a high removal efficiency for NOM and DBP from drinking water. Therefore, TiO_2_ nanoparticles are a promising candidate for the removal of contaminants from drinking water [150]. Rind, et al. [151] studied the use of titanium oxide–magnetic iron oxide nanoparticles as a coating material for kaolinite clay (KC/TiO_2_-Fe_3_O_4_) as a new adsorbent material for the removal of As(III) from drinking water. The results of the authors’ study show that the synthesized KC/TiO_2_-Fe_3_O_4_ exhibited a high adsorption capacity of 462.0 mg g^−1^ for As(III) from drinking water. Based on the results of this study, the synthesized composite KC/TiO_2_-Fe_3_O_4_ is a promising and efficient adsorbent material for As(III) removal from drinking water [151]. Khalid, et al. [152] studied the application of poly(vinyl alcohol) (PVA) membranes incorporated with dopamine-coated titanium oxide nanoparticles for the removal of microbes, dissolved solids, and suspended pollutants from water. The results of the authors’ study show that the synthesized PVA–dopamine-coated TiO_2_ membrane was capable of removing up to 80% of the microbes found in water, with a great removal efficiency for suspended pollutants. Hence, coating membranes with TiO_2_ nanoparticles is an efficient technique for increasing the efficiency of the membrane towards the removal of contaminants from water [152]. Parvizian, et al. [153] investigated the use of TiO_2_ nanoparticles in the synthesis of a hybrid nanofiltration (NF) membrane for the removal of Cu(NO_3_)_2_ from water. The results of the authors’ study show that the synthesized NF membrane was capable of removing up to 79% of the Cu(NO_3_)_2_ found in water. Hence, the incorporation of TiO_2_ nanoparticles into NF membranes is very efficient for enhancing the removal efficiency towards contaminants in drinking water [153]. Lal, et al. [154] synthesized Nd-doped TiO_2_ nanoparticles via a sol–gel ultrasonication process for the photo-catalytic degradation of methylene blue (MB) dye in water under ultraviolet irradiation and solar light. The results of the authors’ study show that the photodegradation efficiency of Nd-doped TiO_2_ towards MB dye was 99.11% under ultraviolet radiation and 96.42% under solar light. Hence, TiO_2_-based photocatalysts are efficient materials that can be used in the remediation of drinking water from dyes and contaminants [154]. Valadez-Renteria, et al. [155] investigated the use of CuS/TiO_2_ (CuST) nanoparticles in the degradation of 4-chlorophenol (4-CP) contaminants from drinking water. The results of the authors’ study show that the CuS/TiO_2_ powders produced a maximum degradation percentage of 92% for 4-chlorophenol (4-CP) under UV–VIS irradiation. Consequently, CuS/TiO_2_ powders are outstanding materials that show great promise for the removal of contaminants from drinking water [155]. Syahin Firdaus Aziz Zamri and Sapawe [156] studied the application of titanium dioxide nanoparticles (TiO2) in the removal of the inorganic pollutant phenol from water. The results of the authors’ study showed that almost all of the phenol was degraded in the water. Hence, TiO_2_ nanoparticles are efficient candidates that can be used in the removal of inorganic contaminants from drinking water [156]. Kazemi, et al. [157] studied the application of photocatalytic polyethersulfone (PES) membranes that were incorporated with nano zerovalent iron@TiO_2_ (NZVI@TiO_2_) photocatalytic nanoparticles in the removal of hexavalent chromium (Cr(VI)) from drinking water. The results of the authors’ study showed that the PES membrane containing titanium oxide nanoparticles achieved a high Cr(VI) removal from water (approximately 100%). As a result, titanium oxide-based membranes are highly effective materials in the removal of contaminants from drinking water [157]. Bindhu, et al. [158] synthesized fluorine- and tin-doped TiO_2_ nanoparticles (Sn–F/TiO_2_ nanoparticles) for the degradation of methylene blue (MB) dye from water under visible and UV light irradiation. The results of the authors’ study showed that the Sn–F/TiO_2_ nanoparticles were efficient in the degradation of MB dye from water. In addition, the nanoparticles exhibited high antibacterial and antifungal action by preventing the growth of bacteria and fungi that were found in drinking water. Therefore, titanium oxide-based nanoparticles are remarkable materials for the elimination of pollutants from drinking water [158]. Gomez-Polo, et al. [159] investigated the application of Cr- and N-doped TiO_2_ anatase nanoparticles (N-TiO_2_, Cr-TiO_2_, and Cr/N-TiO_2_) in the removal of methyl orange dye from water. The results of the authors’ study show that the CR-doped titanium oxide nanoparticles had a higher adsorption capacity for methyl orange and a better antibacterial effect. Hence, titanium oxide-based materials are very efficient in the removal of dyes from water, and can be efficiently used in drinking water purification technologies [159]. Sadeghpour, et al. [160] studied the use of MnFe_2_O_4_/TiO_2_/GO and MnFe_2_O_4_/TiO_2_/g-C_3_N_4_ spinel ferrite nanoparticles in the removal of arsenic from drinking water. The results of the authors’ study demonstrated that MnFe_2_O_4_/TiO_2_/GO and MnFe_2_O_4_/TiO_2_/g-C_3_N_4_ had adsorption capacities of 331 (mg/g) and 332 (mg/g), and removal efficiencies of 98.89% and 99.01%, respectively, for arsenic. According to the results of this study, MnFe_2_O_4_/TiO_2_/GO and MnFe_2_O_4_/TiO_2_/g-C_3_N_4_ are very efficient adsorbents for arsenic elimination from drinking water systems [160]. Zimbone, et al. [161] studied the application of TiO_2_ nanoparticles on the removal of methylene blue dye (MB) and *Escherichia coli* bacteria found in drinking water. The results of the authors’ study show that TiO_2_ nanoparticles exhibited a high removal efficiency against MB dye and a high antibacterial effect towards *Escherichia coli* bacteria. Therefore, TiO_2_ nanoparticles are very efficient materials that can be applied in the removal of contaminants from drinking water [161].

In developing nations, the threat posed by pathogenic diseases continues to endanger people’s lives and health. To provide clean drinking water, a disinfection technique that is effective, economical, and resource-rich is urgently required. Liang, et al. [162] synthesized a novel Ti^3+^-doped TiO_2_ nanoparticle decorated on a ceramic disk filter (Ti^3+^/TiO_2_@CDF) for the disinfection of drinking water. The results of the authors’ study show that the Ti^3+^/TiO_2_@CDF exhibited a high disinfection efficiency for *E. coli* and *S. aureus* bacteria found in drinking water. Hence, Ti^3+^/TiO_2_@CDF is a very efficient material that can be used in the drinking water disinfection process to ensure safe drinking water consumption [162]. Gora, et al. [163] studied the use of TiO_2_ nanomaterials in the removal of natural organic matter (NOM) from drinking water. The results of the authors’ study showed that TiO_2_ nanomaterials were capable of removing NOM from drinking water. Hence, TiO_2_ nanomaterials are efficient materials for drinking water purification technology applications [163]. Table 10 shows a summary of TiO_2_-nanoparticle-based material applications in water purification.

#### 5.2.2. Zinc Oxide Nanoparticles for Drinking Water Purification Processes

**Figure 20:**
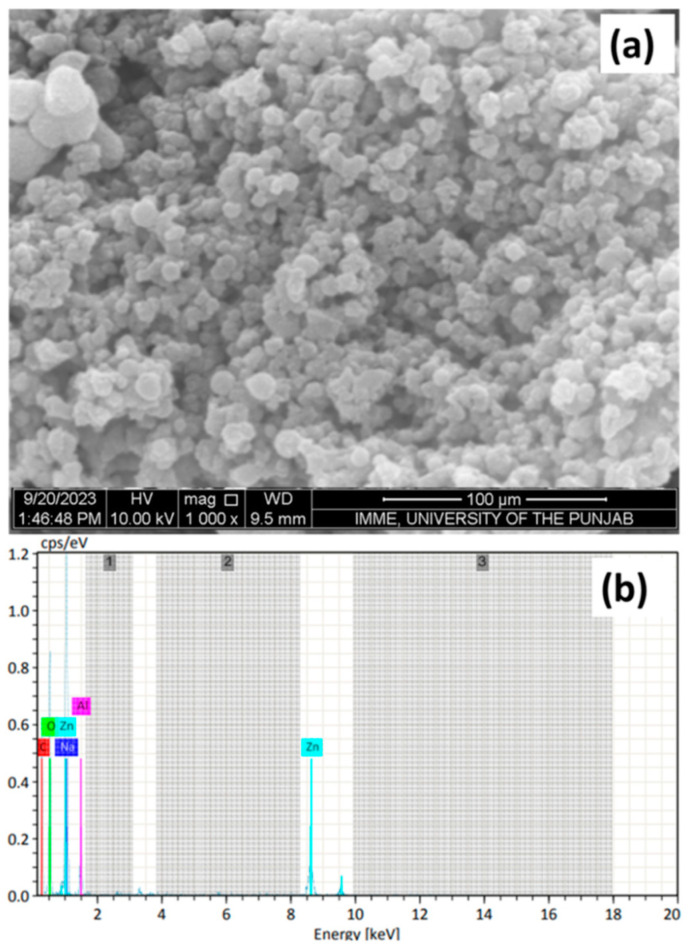
ESEM image of ZnO nanoparticles (**a**) and EDX spectrum of ZnO nanoparticles (**b**) [184].

Zinc oxide nanoparticles (ZnO NPs) have emerged as a promising material for drinking water purification processes owing to their unique properties that enables their efficient usage as a contaminant removal material in drinking water. Figure 20 shows an ESEM image and EDX spectrum of ZnO nanoparticles.

By looking at Figure 20b it is clear that from the EDX analysis there are strong signals displayed that show the presence of zinc and oxygen atoms that exist as a solid in the oxide form. Hence, it can be concluded from the EDX of the ZnO nanoparticles that prominent peaks of 78.32% zinc and 12.78% oxygen are present in the material [184].

The superb properties that ZnO nanoparticles exhibit include the following: great antimicrobial activity, high photocatalytic degradation ability, large surface area, high adsorption capacity, and great biocompatibility. ZnO nanoparticles exhibit exceptional antimicrobial activity against drinking water waterborne pathogens; this is owed to the remarkable photocatalytic activity of ZnO NPs. During the photocatalytic activity of ZnO NPs, a hole and an electron pair are formed (Equation (1)). This photocatalytic activity of ZnO NPs results in water splitting into OH and H^+^ (Equation (2)). Furthermore, the electron produced from the photo-activation reacts with oxygen resulting in superoxide anions (Equation (3)).

(1) $$\mathrm{ZnO}\;\overset{hv}{\to }\;e^{-}+h^{+}$$

(2) h++H2O →OH·+H+

(3) $$e^{-}+O_{2}\to \cdot {O_{2}}^{-}$$

**Figure 21:**
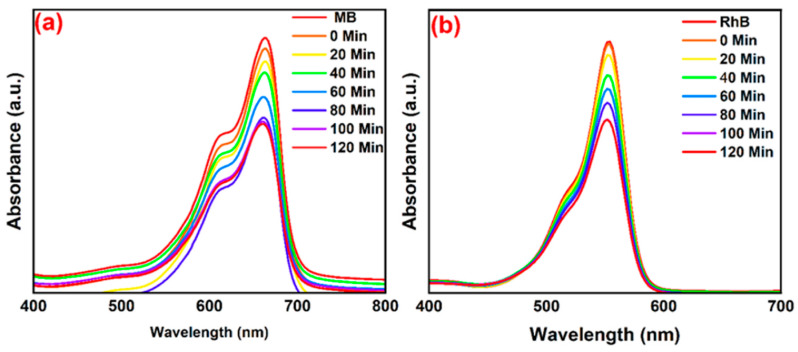
Photolysis activity for the degradation of the MB (**a**) and RhB (**b**) dyes using Ag-doped ZnO/MgO-NCP [185].

The resulting free radicals then react with other organic and inorganic molecules to produce more free radicals, such as H_2_O_2_, or they may alter the chemical structure of the molecule they come into contact with. This explains how ZnO nanoparticles are capable of degrading the organic and inorganic dyes that are found in drinking water. Owing to the remarkable properties that ZnO exhibits, several studies have focused on the application of ZnO NPs in drinking water purification processes. The quality of clean and safe drinking water decreases with the increase in industrial and human activities. Hence, harmful toxins and pathogens must be removed to ensure clean and safe drinking water consumption. In the nano-technological framework, it has been proven that ZnO nanoparticles are a potential material that can be used in the purification of drinking water. Singh, Nath Tripathi, Reddy Prasad and Sunil [147] investigated the application of ZnO nanoparticles as an antimicrobial material for the drinking water purification process. The results of the authors’ study show that ZnO nanoparticles efficiently eliminated microbial pathogens from water. Hence, ZnO NPs are efficient materials that can be used as microbial disinfectants during drinking water treatment processes [147]. Shekofteh Narm, et al. [185] examined the use of a silver-doped zinc oxide/magnesium oxide nanocomposite (Ag-doped ZnO/MgO-NCP) for the degradation of methylene blue (MB) and rhodamine B (RhB) dyes from water. The authors’ also studied the effect of UV irradiation on the degradation of the MB and RhB dyes; see Figure 21.

By looking at Figure 21, which shows the effect of UV irradiation on the degradation of the MB and RhB dyes, it can be concluded that the degradation percentage of the MB and RhB dyes were approximately 33% and 31%, respectively. According to the results of this study, the silver-doped zinc oxide/magnesium oxide nanocomposite is a promising candidate that can be utilized in the removal of the MB and RhB dyes from water [185].

**Figure 22:**
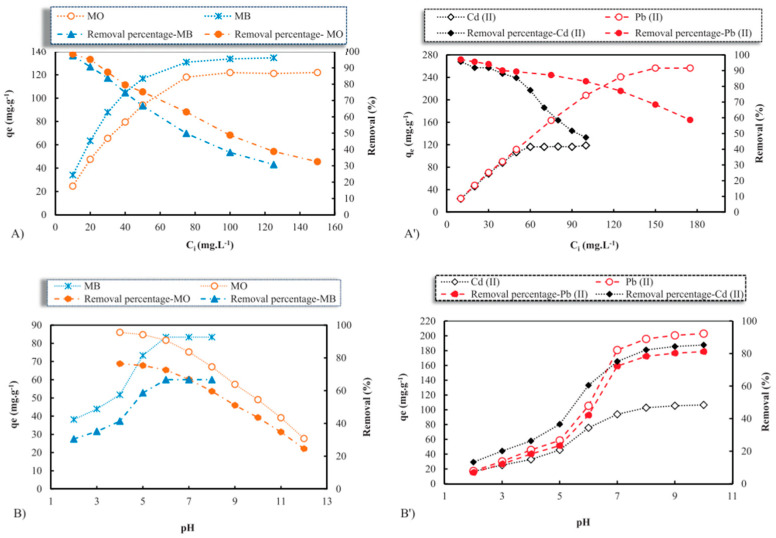
Effect of the initial solution concentrations (**A**) and pHs (**B**) on the dyes and cations as well as the removal (%) by the synthetic nanocomposite GO-ZnO (initial conditions: 25 (mL) of the MB, MO, and Cd^2+^ (50 mg·L^−1^) and Pb^2+^ (100 mg·L^−1^) adsorbates; 10 (mg) of the adsorbent) [35].

Hosseinkhani, Hamzehlouy, Dan, Sanchouli, Tavakkoli and Hashemipour [35] investigated the use of a graphene oxide–zinc oxide (GO-ZnO) nanocomposite as an adsorbent for methylene blue (MB), methyl orange (MO), Cd^2+^, and Pb^2+^ contaminants from water. The authors examined the effect of changing the initial solution concentrations and pHs on the dyes and cations as well as the removal (%) by the synthetic nanocomposite GO-ZnO, as shown in Figure 22.

**Figure 23:**
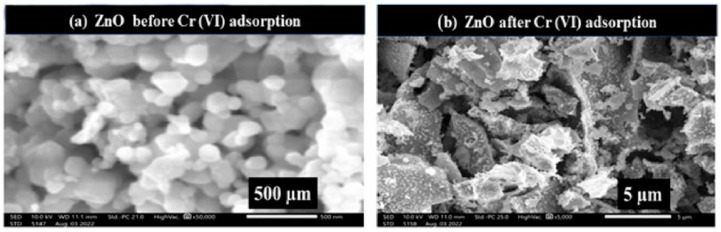
SEM images of (**a**) ZnO before chromium adsorption, (**b**) ZnO after chromium adsorption [186].

By looking at Figure 22, it can be concluded that the highest adsorption capacities reached for methylene blue (MB), methyl orange (MO), Cd^2+^, and Pb^2+^ were 99.00, 125, 121.95, and 277.78 mg/g, respectively, using the GO-ZnO nanocomposite. Based on the results of this study, the GO-ZnO nanocomposite is a remarkable adsorbent for pollutant dyes from water [35]. Jyoti, et al. [186] examined the use of ZnO nanoparticles in the removal of hexavalent chromium (Cr(VI)) from water. The authors’ studied the SEM images of ZnO before and after chromium adsorption; see Figure 23.

By looking at Figure 23, it is clear that from the SEM image before chromium adsorption (Figure 23a), ZnO particles had a random, consistent, regular, and clear crystalline structure. However, after Cr(VI) adsorption (Figure 23b) it can be seen that there is trapped hexavalent chromium present on the surface of ZnO in the form of aggregated clump-like structures. Thus, according to results of this study, ZnO nanoparticles can be applied successfully in the removal of hexavalent chromium from water [186].

The excessive presence of phosphite in the aquatic environment poses severe health concern for humans. Therefore, the removal of phosphite from drinking water is crucial. Liu, et al. [187] investigated the usage of ZnO nanoparticles in the phototransformation of phosphite found in water to phosphate under solar irradiation. The results of the authors’ study showed that the use of ZnO NPs induced the indirect phototransformation of phosphite to phosphate. In addition, the phototransformation process reaction rate increased with the increase of ZnO NP concentration. According to the results of this study, nanophotocatalysts such as ZnO NPs that are released into water environments can act as photosensitizers that transform phosphite to phosphate, thereby mitigating the toxicity of phosphite in the water environment [187]. Hexavalent chromium is regarded as a human carcinogen and its elimination from drinking water is a must. Jyoti, Singh, Das and Srivastava [186] studied the usage of the following adsorbents: chitin, chitosan, zinc oxide, and ZnO–chitosan nanocomposites in the removal of chromium (VI) from water. The results of the authors’ study showed that the maximum adsorption capacity of chromium (VI) was shown by the ZnO–chitosan nano-biocomposite (ZnO-CS NC). The maximum adsorption capacity and efficiency of chromium (VI) reached by ZnO–chitosan were 69.5 mg/g and 96.5%, respectively. Furthermore, ZnO nanoparticles demonstrated great antibacterial activity in water. Therefore, ZnO nanoparticle-based materials are efficient candidates that can be applied successfully in the removal of heavy metal ions from drinking water [186]. Sharma, et al. [188] investigated the use of biosynthesized ZnO NPs in the degradation of methylene blue (MB) dye from water under UV light. The results of the authors’ study revealed that 99% of the MB dye was successfully degraded. Hence, ZnO NPs can be successfully applied as photocatalysts for the degradation of dyes that are found in drinking water. Furthermore, ZnO NPs have demonstrated outstanding antibacterial and antifungal efficacies. Therefore, ZnO NPs are exceptional materials with outstanding properties that can be efficiently applied in the treatment of drinking water [188]. Saqib, et al. [189] studied the application of zinc oxide (ZnO) nanostructures for the degradation of methylene blue dye from drinking water. The results of the authors’ study showed that zinc oxide nanoparticles demonstrated a high photocatalytic activity during the degradation of methylene blue dye under visible light. Hence, zinc oxide NPs are efficient materials that can be successfully applied in the removal of organic pollutants from drinking water [189]. George, et al. [190] synthesized copper-doped zinc oxide nanoparticles via a hydrothermal method, which were tested as adsorbents for methylene blue (MB) dye removal from drinking water. The results of the study revealed that the synthesized copper-doped zinc oxide nanoparticles demonstrated a high degradation efficiency towards methylene blue dye and great antibacterial activity against microbial pathogens in water. Hence, ZnO nanoparticle-based materials can be successfully applied in photodegradation and antibacterial applications for drinking water treatment processes [190]. Mondal, et al. [191] synthesized pristine- and nitrogen (N)-doped zinc oxide (ZnN_x_O_1−x_, x = 0, 0.005, 0.01, and 0.02) nanoparticles (NPs) via a chemical co-precipitation approach for the photodegradation of rhodamine B (RhB) from water. The results of the authors’ study showed that the highest photocatalytic activity towards the degradation of rhodamine B (RhB) was achieved by ZnN_0_._01_O_0_._99_ NPs. Hence, zinc oxide NP-based photocatalysts can be efficiently applied in the removal of dyes from drinking water [191].

In recent years, pharmaceutical contaminants have emerged as a new global environmental issue that must be addressed, owing to the potential hazards that they possess to both the environment and human health. Gayathri, et al. [192] investigated the use of ZnO nanoparticles as a photocatalyst for the degradation of the following pharmaceutical contaminants that are found in water: sulfamethoxazole (SMX), tetracycline (TCT), chloroquine (CLQ), and diclofenac (DCF) under sunlight. The results of the authors’ study showed that ZnO NPs resulted in a degradation of approximately 65% for SMX, 61% for CLQ, 55% for DCF, and 51% for TCT within 30 min under solar irradiation. According to the results of this study, ZnO NPs are very effective materials for the degradation of pharmaceuticals that are found in water. Hence, they can be used efficiently for drinking water purification processes [192]. Al Aqad and Basheer [193] synthesized a reduced graphene oxide nanosheet (RGO) with ZnO nanocomposite (Zno/RGO) for the photocatalytic degradation of basic blue dye in water. The results of the authors’ study show that the maximum photocatalytic degradation reached for the basic blue dye was 98%, using the ZnO/RGO nanocomposite. Hence, ZnO nanoparticle-based materials are efficient candidates for the degradation of dyes that are found in drinking water [193]. Sasi, et al. [194] studied the application of ZnO NPs in the degradation of dyes from drinking water. The results of the authors’ study revealed that ZnO NPs were very effective in the photocatalytic degradation of the dyes from drinking water. In addition, ZnO NPs exhibited a high antimicrobial activity against pathogens that were found in the water. Hence, ZnO NPs are potential candidates for drinking water pollutant remediation processes [194]. Gu, et al. [195] fabricated zinc oxide nanoparticles (ZnO-NPs) and investigated their application as an adsorbent for the removal of Cr^3+^ from drinking water. The results of the authors’ study revealed that the maximum adsorption capacity for Cr^3+^ ions reached was 88.547 mg/g. According to the results of this study, ZnO NPs are potential candidates that can be applied in the removal of heavy metal ions from drinking water [195]. Choina, et al. [196] studied the usage of ZnO NPs in the removal of the following pharmaceuticals from drinking water: tetracycline and ibuprofen. The results of the authors’ study revealed that ZnO NPs were very efficient in the removal of tetracycline and ibuprofen from drinking water [196]. Excess fluoride concentration in drinking water possess a severe risk to the health of human beings as well as the environment. Noor, et al. [197] prepared a zinc oxide-based nanocomposite GO/CoFe_2_O_4_/ZnO for the removal of fluoride ions from drinking water. The results of the authors’ study showed that the maximum removal efficiency of fluoride ions reached by the GO/CoFe_2_O_4_/ZnO nanocomposite was 94.5%. According to the results of this study, zinc oxide-based nanocomposites are potential adsorbents that can be used efficiently in the removal of fluoride from drinking water [197]. Singh, et al. [198] synthesized ZnO NPs via citrus lemon leaf extract as a sustainable and cost-effective approach, and investigated their application in the removal of reactive green-19 (RG-19) dye and bacteria from drinking water. The results of the authors’ study showed that ZnO NPs exhibited a high photocatalytic degradation of reactive green-19 (RG-19) dye with approximately 92% degradation efficiency. Furthermore, ZnO NPs demonstrated a high antibacterial activity against two pathogenic Gram-positive (*Bacillus subtilis*) and Gram-negative (*Escherichia coli*) bacteria. Thus, this study presents an effective and sustainable ZnO NP-based platform for drinking water purification [198]. Pillai, et al. [199] synthesized Al-doped ZnO nanoparticles via the sol–gel method for the defluoridation of drinking water. The results of the authors’ study showed that Al-doped ZnO NPs successfully removed fluoride ions from water. Hence, Al-doped ZnO NPs could be a promising adsorbent for an efficient defluoridation process for drinking water purification [199]. Motshekga, et al. [200] synthesized a composite of silver–zinc oxide nanoparticles supported on bentonite clay (Ag/ZnO-clay composite) for use as an antibacterial substance in drinking water. The results of the authors’ study revealed that the Ag/ZnO-clay composite had a high antibacterial activity against Gram-negative *Escherichia coli* bacteria and Gram-positive *Enterococcus faecalis* bacteria. According to the results of this study, the Ag/ZnO-clay composite is a promising bactericide that can be applied in the removal of microbes in drinking water [200].

Antimicrobial nano-sized zinc oxide (ZnO) materials have been utilized as environmentally friendly nanocomposites for drinking water purification applications owing to their superb properties. Atta, et al. [201] investigated the use of a ZnO nanocomposite as an adsorbent for methylene blue dye from water. The results of the authors’ study demonstrated that the ZnO nanocomposite achieved a high adsorption removal rate for MB dye reaching 3000 mg/L from water. Hence, ZnO-based nanocomposites are potential candidates for the removal of organic dyes from drinking water [201]. Dilawar, et al. [202] synthesized an Ag/Mn–ZnO nanomaterial using a one-pot hydrothermal approach and analyzed its photocatalytic activity against organic dyes and bacteria that are found in water. The results of the authors’ study revealed that the Ag/Mn–ZnO nanomaterial successfully photocatalytically degraded up to 70% of both of the following dyes: methyl orange and alizarin red from water. In addition, a high anti-bacterial activity against *E. coli* bacterium was observed. Consequently, the synthesized Ag/Mn–ZnO nanomaterial is an efficient tool for the remediation of drinking water from dyes and bacteria contaminants [202]. Whang, et al. [203] prepared Ag-doped ZnO nanoparticles (Ag/ZnO) for the photocatalytic degradation of methylene blue (MB) dye from water. The results of the authors’ study showed that Ag/ZnO nanoparticles were very efficient in photocatalytically degrading MB dye from water. Hence, zinc oxide nanoparticle-based materials are effective candidates that can be successfully applied for the removal of organic dyes from drinking water [203]. Based on all of the above studies, zinc oxide NP-based materials are effective candidates for the removal of several types of contaminants from drinking water. Table 11 shows a summary of ZnO nanoparticle-based material applications in water purification.

#### 5.2.3. Silver Nanoparticles (Ag NPs) for Drinking Water Purification

**Figure 24:**
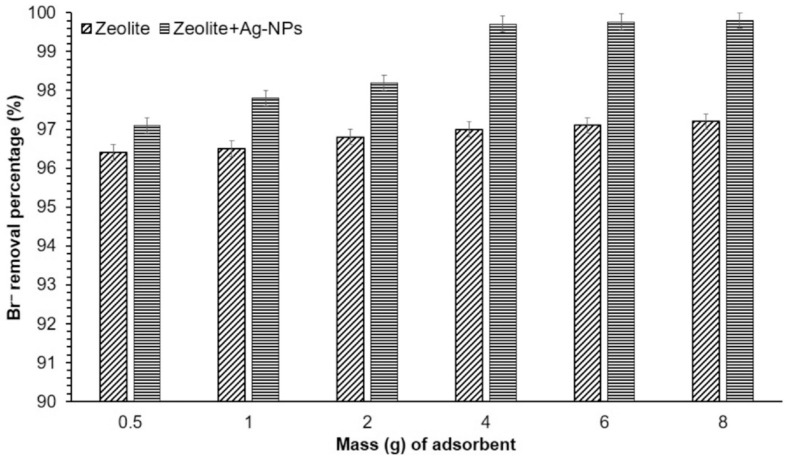
Bromide ion removal efficiencies of zeolite and zeolite+Ag-NPs at different adsorbent dosages and constant temperature and time [228].

Silver nanoparticles (AgNPs) have remarkable properties, including a high antibacterial effect, high photocatalytic activity, and long-lasting photocatalytic and antibacterial effects, which enable their usage in drinking water purification processes. At the present time, silver NPs are widely applied in the removal of pathogenic microorganisms, heavy metals, and organic pollutants from drinking water. Several studies have focused on the application of silver NPs in the removal of contaminants from drinking water owing to their unique properties. Al-Rawajfeh, et al. [228] examined the use of a silver nanoparticle (Ag-NP)–zeolite composite as an adsorbent for bromide ions (Br^−^) from water. The authors’ studied the bromide ion removal efficiencies of zeolite and zeolite+Ag-NPs at different adsorbent dosages and constant temperature and time; see Figure 24.

As can be seen from Figure 24, the silver nanoparticle (Ag-NP)–zeolite composite had a higher Br^-^ removal efficiency at all adsorbent dosages compared to only zeolite. Furthermore, the highest Br^-^ removal efficiency reached was approximately 100% using 8 g of silver nanoparticle (Ag-NP)–zeolite composite. In addition, as the adsorbent dosage increases, the bromide ion removal efficiency also increases for both zeolite and zeolite+Ag-NPs. According to the results of this study, the integration of Ag-NPs into zeolite offers a promising solution for sustainable and multifunctional water treatment solutions [228]. Soliman, et al. [229] investigated the use of activated carbon (AC) that was decorated with silver nanoparticles (Ag NPs) in the removal of bacteria and inorganic ions from drinking water. The results of the authors’ study showed that Ag-loaded AC exhibited exceptional antibacterial activity against Escherichia coli and Staphylococci bacteria. Furthermore, Ag-loaded AC efficiently removed some inorganic disinfection by products (DBPs) including chlorate, iodate, chlorite, and bromate. According to the results of this study, AC-Ag is a potential candidate for the removal of microbial pathogens and heavy metal ions from drinking water [229]. Neme, et al. [230] impregnated silver nanoparticles (Ag NPs) on activated carbon (AC), which were tested for the removal of bacteria from water. The results of the authors’ study showed that AC-Ag NPs removed 100% of the *E. coli* bacteria that were found in water. Hence, silver NP-based materials are very efficient in the removal of bacteria from drinking water, and can be used in drinking water purification processes [230]. Nuthalapati, et al. [231] synthesized an innovative nanocomposite material (Alg@Ag/PU) by modifying silver nanoparticles (Ag NPs) with alginate (Alg) and coating them on a polyurethane sponge (PU) surface for heavy metal ion removal from water. The results of the authors’ study demonstrated that the the Alg@Ag/PU nanocomposite displayed an excellent removal efficiency for heavy metal ions from water. Consequently, silver NP-based materials are excellent candidates that can be applied in drinking water purification processes for the removal of heavy metal ions [231]. Wankar, et al. [232] fabricated a silver–chitosan (Ag-CH) nanocomposite hydrogel for the removal of hexavalent chromium (Cr (VI)) from drinking water. The results of the authors’ study showed that the Ag-CH hydrogel exhibited a high percentage removal of Cr (VI), reaching 83%, from water. Hence, silver nanoparticle-based composite materials are effective candidates for the removal of heavy metal ions from drinking water, and can be applied effectively in drinking water purification processes [232]. Luukkonen, et al. [233] synthesized a geopolymer–bentonite composite foam with silver nanoparticles and investigated its application in the removal of bacteria from drinking water. The results of the authors’ study showed that the synthesized geopolymer had a high inactivation efficiency against *Escherichia coli* and intestinal enterococci bacteria that were found in drinking water. Therefore, silver NP-based geopolymers are powerful materials that can successfully kill bacteria that are found in drinking water [233].

The continuous search for safe, cost-effective, and efficient materials for the removal of pathogens from drinking water is increasing over time. Ahsan, et al. [234] investigated the use of silver nanoparticle-embedded filter paper for the elimination of antibiotic-resistant bacteria from drinking water. The results of the authors’ study showed that Ag NPs exhibited a high bactericidal activity (approximately 100%) for the antibiotic-resistant *E. coli* bacteria that were found in drinking water. As a result, AgNP-embedded filter paper is an effective approach for the disinfection of drinking water from harmful bacteria [234]. The photocatalytic inactivation of microorganisms including viruses and bacteria that are found in drinking water is a promising approach that has been increasingly applied in recent decades. Liga, et al. [235] investigated the use of photocatalytic silver-doped titanium dioxide nanoparticles (Ag/TiO_2_) for inactivating the bacteriophage MS2 in drinking water. The results of the authors’ study showed that Ag/TiO_2_ NPs were capable of inactivating the bacteriophage MS2 with a high efficiency. Hence, Ag NPs are efficient materials for the removal of pathogenic microbes from drinking water [235]. Yang, et al. [236] investigated the use of a GO-Ag catalytic membrane in the removal of bisphenol A (BPA) from water. The results of the authors’ study showed that the graphene oxide/silver NP-based catalytic membrane effectively decomposed greater than 99.4% of the BPA from water. Furthermore, the GO-Ag catalytic membrane exhibited a high antibacterial activity in water. Hence, the immobilization of Ag NPs in catalytic membranes is an efficient technique for the removal of contaminants from drinking water [236]. Mohsentabar, et al. [237] studied the application of an Ag/ZnO nanocatalyst that was synthesized via a hydrothermal method in the photodegradation of acetaminophen in water. The results of the authors’ study showed that the Ag/ZnO nanocatalyst exhibited a high removal efficiency of up to 94% for acetaminophen from water. Hence, Ag NP-based nanocatalysts are efficient materials that can be applied in drinking water purification technologies [237]. Rajanandkumar [238] investigated the use of Ag NPs in the removal of bacterial microorganisms including *E. coli* bacteria from drinking water. The results of the author’s study revealed that Ag NPs exhibited a high antibacterial effect towards *E. coli* bacteria resulting in their removal. Hence, Ag NPs are potential candidates for the purification of drinking water from bacterial microorganisms [238].

The existence of *Escherichia coli* in water causes severe pollution to drinking water and several health and environmental issues. At the present time, a wide range of materials are being investigated for the removal of contaminants from drinking water. Bilal, et al. [239] investigated the use of silver nanoparticles (Ag NPs) that were impregnated with various support materials including zeolite, activated carbon, and magnesium oxide for the inhibition of bacteria in drinking water. The results of the authors’ study show that the Ag NP-supported materials successfully inhibited all the bacteria that were found in drinking water. In addition to their antibacterial effects, the synthesized catalysts exhibited remarkable methyl orange azo dye reduction activity. Furthermore, the highest reduction rate of dye was achieved by MgO-impregnated silver NPs catalysts for methyl orange dye, with a reduction rate constant of 7.48 × 10^−3^ s^−1^. According to the results of this study, supported Ag NPs materials have the potential to be applied in the elimination of microbial and organic contaminants from drinking water [239]. Wang, et al. [240] synthesized an innovative porous and renewable aerogel by using collagen fibers (CFs), Fe^3+^ ions with gallic acids, and modified silver nanoparticles (AgNPs/Fe@CF) for the purification of water from bacteria and dyes. The results of the authors’ study showed that the Ag NP-incorporated aerogel exhibited a high antibacterial activity against tetracycline-resistant *E. coli* and methicillin-resistant Staphylococcus aureus. In addition, the AgNPs/Fe@CF aerogel demonstrated a high catalytic degradation efficiency for five distinct antibiotics with greater that 90% degradation efficiency. Moreover, the fabricated aerogel showed a high removal rate for the following heavy metals: Cr (VI), Ni (II), and Pb (II). Based on the results of this study, Ag NP-based aerogels are potential candidates that are capable of removing several contaminants from water and can be successfully applied in drinking water purification processes [240]. Gong, et al. [241] synthesized a plasmonic Ag NP-decorated MIL-101(Fe) hybrid (Ag/MIL-101(Fe)) via a photodeposition approach for the removal of peroxymonosulfate (PMS) and bisphenol A (BPA) from water. The results of the authors’ study showed that the synthesized Ag/MIL-101(Fe) exhibited a high photocatalytic activity against PMS and BPA. Hence, Ag NP-based materials are very effective in the removal of pollutants from drinking water [241].

The presence of any trace concentrations of mercuric ions (Hg^2+^) in drinking water causes severe health effects in human beings; therefore, its removal from drinking water is a must. Sumesh, et al. [242] investigated the use of silver NPs that are protected by mercaptosuccinic acid (MSA) and supported on activated alumina for the elimination of mercuric ions (Hg^2+^) that are found in contaminated water. The results of the authors’ investigation revealed that the synthesized Ag@MSA exhibited a high removal rate for Hg^2+^ from water. Hence, Ag@MSA can be successfully used in the removal of mercuric ions from drinking water [242]. Biswas and Bandyopadhyaya [243] investigated the use of silver nanoparticles (Ag-NP) that were impregnated on the external surface of activated carbon (AC) granules (Ag-AC hybrid) for the removal of bacteria from drinking water. The results of the authors’ study showed that the synthesized Ag-AC hybrid exhibited a high removal rate for *Escherichia coli* bacteria, reaching a 100% removal rate, from drinking water. Hence, Ag NP-based hybrid materials are very effective in the removal of pollutants and bacteria from drinking water [243]. Huang, et al. [244] investigated the use of Ag/ZnO nanocomposites as a coating material for ceramic water filters for the removal of bacterial from drinking water. The results of the authors’ study showed that the use of Ag NPs in coating the ceramic filters increased the removal efficiency of the filter for *Escherichia coli* (*E. coli*) bacteria. Hence, Ag NP-based filters are exquisite materials that can be used effectively for pathogen and bacteria removal from drinking water [244]. Table 12 shows a summary of Ag nanoparticle-based material applications in water purification.

#### 5.2.4. Iron Nanoparticle (Fe NPs) Applications in Drinking Water Purification Processes

Iron nanoparticles (Fe NPs), are a cutting-edge nanotechnology that could completely transform the drinking water purification process. Iron nanoparticles are characterized by having a large surface area, high reactivity, and remarkable magnetic properties, which make them very efficient materials for the removal of a wide range of contaminants from drinking water. Fe NPs can efficiently eliminate heavy metals from drinking water via adsorption and reduction processes. The large surface area of Fe NPs allows them to bind to heavy metal ions in drinking water, and their reductive capability allows them to transform the toxic metal ions into less harmful forms. This is very beneficial for the elimination of heavy metals that are extremely harmful to health, such as lead, mercury, and arsenic. Furthermore, Fe NPs can also be applied in the degradation of organic pollutants from drinking water using Fenton-like reactions. During the Fenton-like reactions, hydroxyl radicals (•OH) are generated, which are highly reactive and are capable of breaking down the organic pollutants into harmless byproducts. Fe NPs function as catalysts for these reactions by stimulating the production of hydroxyl radicals (•OH) and speeding up the organic pollutants’ degradation process. Moreover, Fe NPs can be applied as an antimicrobial material for the disinfection of drinking water and the elimination of harmful microorganisms. Owing to their large surface area, Fe NPs are capable of interacting with the microbial cells, which causes damage to their cell membranes and leads to microbial cell inactivation. This technique is very efficient against a wide range of bacteria, viruses, and protozoa. In addition to their remarkable abilities in the purification of drinking water, Fe NPs are also relatively inexpensive and easy to produce. This makes them a potentially useful technology in developing nations where it is difficult to obtain clean and safe drinking water.

Owing to these remarkable properties and abilities, several studies have focused on the application of Fe NPs in the removal of contaminants from drinking water.

**Figure 25:**
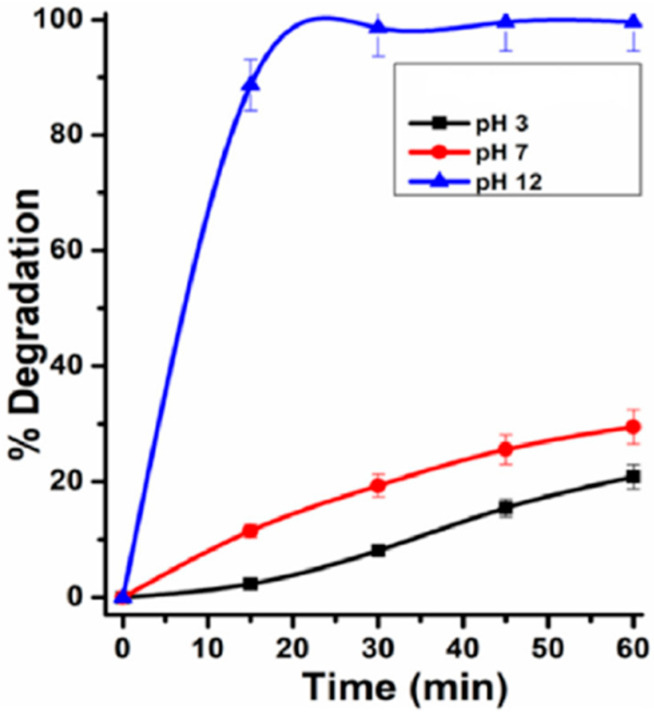
Degradation % of MB using Fe_3_O_4_ NPs at different pH values [262].

Mahlaule-Glory, et al. [262] investigated the use of iron oxide (Fe_3_O_4_) NPs in the removal of methylene blue dye from water. The authors investigated the percentage degradation of MB dye at different pH levels using Fe_3_O_4_ NPs; see Figure 25.

**Figure 26:**
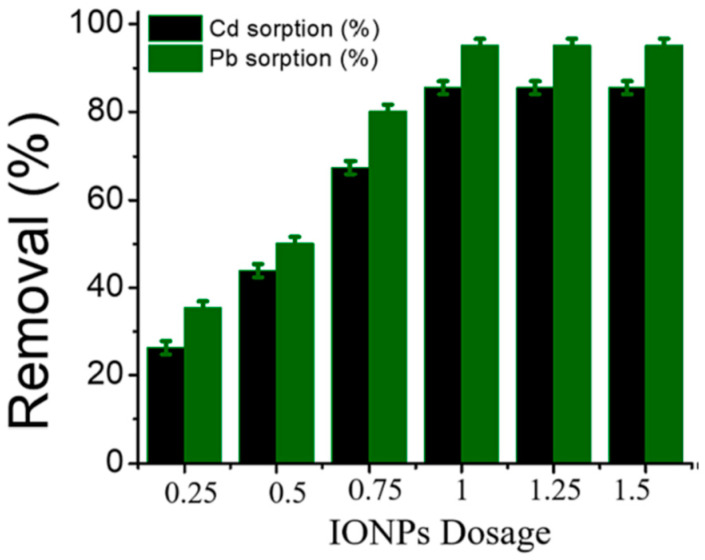
Effect of IONPs dosage (g/L^−1^) on Cd and Pb removal (%) [263].

By looking at Figure 25, it is clearly seen that the maximum degradation % of MB dye reached by using Fe_3_O_4_ NPs is 99% in less than 30 min, at pH 12. This can be owed to the fact that MB dye has a cationic nature that dissociates into negatively charged ions, particularly at pH 12. In addition, the lowest MB dye degradation % performance was achieved at under pH 3. According to the results of this study, Fe_3_O_4_ NPs hold great promise as efficient adsorbents for MB dyes from water, and can be utilized in water treatment systems [262]. Kaleem, et al. [263] studied the use of iron oxide nanoparticles (IONPs) in the removal of lead (Pb) and cadmium (Cd) ions from water. The authors investigated the effect of changing the amount of IONPs on the percentage removal of Pb and Cd ions from water; see Figure 26.

By looking at Figure 26, which describes the effect of IONPs dosage on Cd and Pb removal (%), it is clearly seen that generally speaking as the IONPs dosage increases the percentage removal of Cd and Pb ions also increases. Furthermore, the maximum % removal of Pb reached was 95.11% and 85.43% for Cd at 1.0 g/L of IONPs. In addition, as the IONPs content increased from 1.0 g/L to 1.5 g/L, the percentage removal of both ions stayed the same (95.11% for Pb and 85.43% for Cd). Based on the results of this study, IONPs are very successful in the remediation of Pb and Cd contaminants from water [263]. Pabón Reyes, et al. [264] investigated the use of zerovalent iron nanoparticles (nZVI) in the removal of arsenical As(III) from drinking water, under UV–Vis irradiation (*λ*_em_ = 300–800 nm). The results of the authors’ study show that the use of zerovalent iron nanoparticles (nZVI) efficiently removed up to 92% of the arsenic present in drinking water. Hence, Fe NPs can be successfully used in the removal of heavy metals from drinking water [264]. Zhang, et al. [265] studied the application of nano zerovalent iron (nZVI) for the removal of low concentration of Hg(II) from drinking water. The results of the authors’ study showed that nano zerovalent iron (nZVI) was very efficient in the removal of low Hg(II) concentrations from drinking water. Thus, Fe NPs are a potential candidates for the purification of drinking water [265]. Maamoun, et al. [266] studied the use of magnesium hydroxide-encapsulated iron nanoparticles (nFe^0^@Mg(OH)_2_) in the removal of chromium (Cr(VI)) from drinking groundwater. The results of the authors’ study show that the applied nFe^0^@Mg(OH)_2_ efficiently removed up to 100% of the Cr(VI) present in the groundwater. Hence, Fe NPs can be successfully applied in drinking water purification processes [266]. Asadi Haris, et al. [267] synthesized superparamagnetic iron oxide nanoparticles (SPIONs) via a hydrothermal method and investigated its usage in the form of alginate bead-encapsulated (SPIONs-Alg) nanoparticles in the removal of arsenite, As(III), from drinking water. The results of the authors’ study show that the maximum As (III) removal percentages for SPIONs and SPIONs-Alg were 99% and 90%, respectively. Furthermore, the maximum adsorption capacity for As(III) reached by the two adsorbents were found to be 11.89 mg/g and 240.081 mg/g for SPIONs and SPIONs-Alg, respectively. The results of the authors’ study show that Fe NPs in their different forms can be successfully applied in the removal of heavy metals from drinking water [267]. Bounab, et al. [268] investigated the use of reductive zerovalent iron nanoparticles in the removal of Cr(VI) from water. The results of the authors’ study show that the prepared Fe NPs were successful in the removal of Cr(VI) from water. Hence, Fe NPs are potential candidates that can be applied efficiently in drinking water purification processes [268]. Mahanty, et al. [269] synthesized mycosynthesized magnetic iron oxide (γ-Fe_2_O_3_) nanoparticles (IO-NPs) for the removal of the following heavy metals from water: Pb, Ni, Cu, Zn, Cd, and Cr. The results of the authors’ study showed that the synthesized iron oxide NPs effectively adsorbed 98.91% of Cd, 92.86% of Pb, 94.93% of Ni, 95.92% of Cu, 98.11% of Zn, and 82.12% of Cr from water. According to the results of this study, iron oxide NPs are very effective adsorbents for the removal of heavy metals from water [269].

Drinking water contamination due to pathogens, dyes, and antibiotics poses a serious risk to humans, animals, and other living things. Therefore, it is necessary to fabricate new efficient materials that can be utilized to break down different types of contaminants in drinking water. Mahlaule-Glory, Mapetla, Makofane, Mathipa and Hintsho-MBita [262] investigated the application of iron oxide (Fe_3_O_4_) NPs in the photocatalytic degradation of dyes (methylene blue (MB) dye) and the removal of bacteria (*E. coli* and *S. aureus* bacterial strains) and antibiotics (sulfisoxazole) from drinking water. The results of the authors’ study showed that Fe_3_O_4_ NPs degraded up to 60% of the sulfisoxazole, and efficiently removed the MB dye and the bacteria that were found in water. Hence, Fe NP-based materials can be efficiently used in the removal of several pollutants from drinking water [262]. Munoz, et al. [270] investigated the use of carbon-encapsulated iron nanoparticles (CE-nFe) in the removal of the following antibiotics: diclofenac (DCF), sulfamethoxazole (SMX), and metronidazole (MNZ) from water. The results of the authors’ study showed that the carbon-encapsulated iron nanoparticles (CE-nFe) effectively removed the specified antibiotics from water. Therefore, Fe NP-based materials can be successfully applied in drinking water purification processes [270]. Conde-Cid, et al. [271] studied the application of green zerovalent iron nanoparticles (gnZVIs) in the removal of the antibiotic sulfadiazine (SDZ) from water via adsorption and reduction processes. The results of the authors’ study showed that gnZVIs removed up to 69% of SDZ from water. According to the results of this study, Fe NP-based materials are potential candidates that can be applied in the removal of antibiotics from drinking water [271]. Table 13 shows a summary of Fe nanoparticle-based material applications in water purification.

### 5.3. Nanomaterial-Based Membranes for Drinking Water Purification Processes

**Figure 27:**
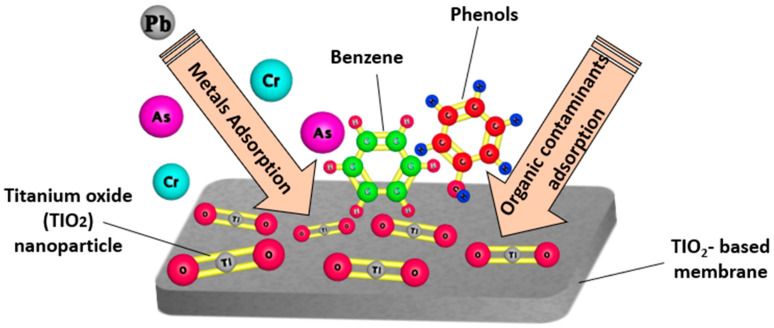
Schematic presentation of the adsorption process of heavy metals and organic water contaminants by a TIO_2_ nanoparticle-based membrane.

Membrane technologies play an essential role in drinking water purification processes by acting like a physical barrier that separates several types of pollutants from drinking water. Figure 27 shows a schematic presentation of the heavy metal and organic water contaminant adsorption process by TIO_2_ nanoparticle-based membranes. The microscopic openings that are found in the membrane allows the water molecules to pass, and prevents the larger contaminant molecules to pass through the membrane openings. The membrane filtration process can be categorized as follows: dead-end filtration and cross-flow filtration. In dead-end filtration, the input water stream containing contaminants flows perpendicular to the membrane surface. After that, all the contaminant solids are collected onto the membrane surface during the filtration process, and then removed via backwashing. On the other hand, in cross-flow filtration, the input water stream is parallel to the membrane surface, and most of the solid pollutants will pass with the water instead of building up on the membrane surface.

The underlying trade-off between membrane permeability and selectivity is a core problem in membrane technology. In addition, the performance of membrane technology is hindered by several factors including high cost, fouling, and high energy consumption. As a matter of fact, the wide range of applications for pressure-driven membrane processes is significantly limited by their high energy consumption. Generally speaking, the efficiency of membrane technology is largely dependent on the material that the membrane is made of. Hence, choosing the membrane material is crucial for the overall drinking water purification process efficiency. There are several types of membranes that can be used in drinking water purification processes. These types can be categorized as follows: microfiltration (MF), ultrafiltration (UF), nanofiltration (NF), reverse osmosis (RO), and electrodialysis (ED) membranes; each type of membrane is applied in the removal of specific types of contaminants. Table 14 shows the application of each type of membrane in the drinking water purification process.

**Figure 28:**
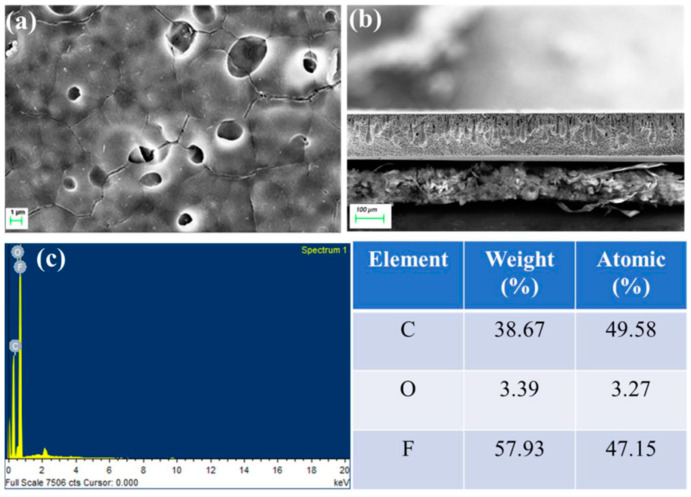
SEM surface image (**a**); cross-section image and EDAX spectra (**b**,**c**) of a SAGO@PVDF membrane [293].

The targeted water quality and the properties of the raw input water determines which membrane method is used. Membrane technology has completely transformed the process of purifying drinking water, offering communities all over the world a sustainable, effective, and safe way to produce clean drinking water. Owing to the remarkable applications of membranes in drinking water purification, nanoparticles have been used in the synthesis of these membranes. Several studies have focused on the use of nanotechnology in the fabrication of membranes for drinking water purification. Islam, et al. [293] synthesized shear-aligned graphene oxide (GO) nanosheet-incorporated polyvinylidene fluoride (PVDF) membranes (SAGO@PVDF) for the removal of methylene blue (MB), rhodamine B (RB), Congo red (CR), and methyl orange (MO) dyes from water. The authors examined the surface morphology and cross-section of the SAGO@PVDF membrane using scanning electron microscopy (SEM); see Figure 28.

**Figure 29:**
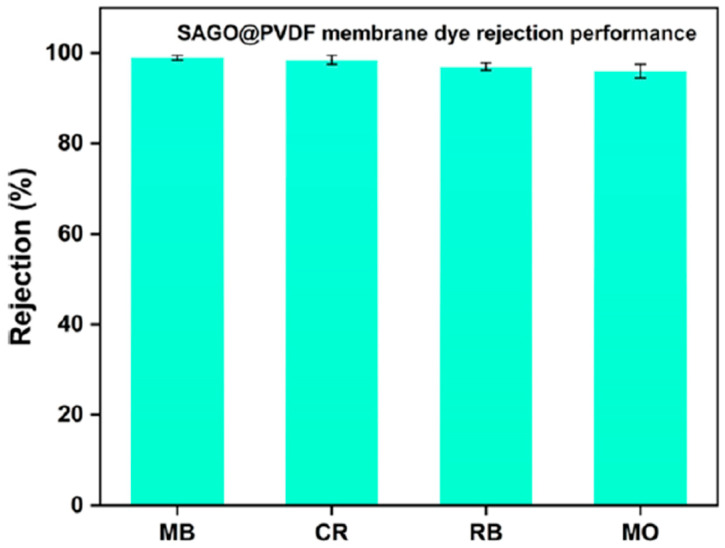
Rejection (%) of MB, CR, RB, and MO dyes using the SAGO@PVDF membrane [293].

By looking at the SEM image in Figure 28a, it is shown that the surface of SAGO@PVDF composite membranes has a continuous, rough, wavy-type structure. In addition, the cross-section view (Figure 28b) illustrates the thickness of 150 mm that is formed by shear-aligning GO sheets to the PVDF matrix. Furthermore, the peaks shown in the EDAX spectra of SAGO@PVDF (Figure 28c) indicate the presence of carbon, oxygen, and fluorine elements. The performance of the SAGO@PVDF composite membranes in the rejection of MB, CR, RB, and MO dyes is shown in Figure 29.

By looking at Figure 29, it is clearly seen that the rejection (%) of all dyes is greater than 95%. Furthermore, the highest rejection (%) achieved by the SAGO@PVDF membrane was 99% for the MB dye. According to the results of this study, the SAGO@PVDF membrane is a remarkable solution for the efficient removal of dyes from water [293].

In many parts of the world, arsenic water pollution is endangering the safety of drinking water. Arsenite, or As(III), is the most challenging type of arsenic to remove from drinking water owing to its neutrality in drinking water. Hence, the fabrication of novel materials that can efficiently remove arsenite from drinking water is essential. Luan, et al. [294] fabricated novel nanocomposite materials (Cu/CNT) by intercalating Cu nanoparticles into carboxylated/hydroxylated carbon nanotubes (CNTs) that were then deposited on top of a polymeric membrane. The membrane was then tested for As(III) removal from drinking water. The results of the authors’ study showed that the as-prepared membrane successfully removed more than 90% of As(III) from drinking water. Thus, the novel Cu/CNT nanocomposite membranes have shown promising potential in the elimination of As(III) from polluted drinking water, and can be successfully applied in drinking water purification processes [294]. Alsalme [295] synthesized a multi-layer graphene oxide hybrid membrane using Fe_3_O_4_@amyloid fibril nanoclusters intercalated into the graphene oxide sheets by a flow-through approach, and investigated the application of the hybrid membrane in the removal of heavy metals from drinking water. The results of the authors’ study showed that the graphene-based hybrid membrane exhibited a high removal efficiency with a value greater than 99.9% for heavy metal ions from drinking water. Hence, graphene-based hybrid membranes can be efficiently used as a tool for the remediation of drinking water from heavy metal ions [295]. Zambianchi, et al. [296] investigated the application of polysulfone-graphene oxide hollow fiber membranes (PSU-GO HFs) for simultaneous adsorption and ultrafiltration abilities for heavy metals including Pb, Cu, and Cr(III) from drinking water. The results of the authors’ investigation showed that the fabricated membrane had a high removal efficiency of Pb, Cu, and Cr(III) from drinking water. Hence, graphene oxide-based membranes are potential candidates that can be efficiently applied for the removal of heavy metal ions from drinking water [296]. Kamarudin, et al. [297] synthesized silver type 1 (AgNPs-1) and type 2 (AgNPs-2) nanoparticles and incorporated them into a polyvinylidene fluoride (PVDF) ultrafiltration membrane in the presence of thermal treatment, and investigated membrane usage in the removal of *E. coli* bacteria from drinking water. The thermal treatment effects were investigated by the authors at temperatures of 25 °C, 40 °C, and 55 °C, which greatly affected the physicochemical properties of the PVDF membrane. The results of the authors’ study showed that the membranes with AgNPs-1 and AgNPs-2 had a high bactericidal performance against *E. coli* with a percentage reduction of 90.38% and 80.77%, respectively. Consequently, membranes with Ag NPs are potential candidates that can be applied in the disinfection of drinking water from harmful bacteria [297]. Chen and Peng [260] synthesized a silver nanoparticle-decorated cellulose nanofibrous (CNF) membrane (Ag/CNF) via an in situ reduction method for the removal of bacteria from drinking water. The results of the authors’ study showed that the Ag/CNF membrane efficiently removed up to 86% of the bacteria found in drinking water. Hence, silver nanoparticle-based membranes can be successfully applied in the disinfection process of drinking water to remove unwanted harmful bacteria [260]. Marrero, et al. [298] fabricated poly(vinylidene fluoride) (PVDF) membranes that were doped with reduced graphene oxide (rGO-PVDF) and were tested for the removal of bacteria (*Escherichia coli* and *Enterococcus faecalis*) from drinking water. The results of the authors’ study showed that the synthesized rGO-PVDF membrane had a high bactericidal efficiency towards the *Escherichia coli* and *Enterococcus faecalis* bacteria that were found in drinking water. Hence, rGO-PVDF membranes are promising candidates that can be successfully applied in the removal of bacteria from drinking water [298].

High fluoride ion (F^−^) levels in drinking water has dangerous effects on human health. Hence, the reduction of fluoride ions is essential to maintain safe drinking water. Yu, et al. [299] synthesized a sulfonated graphene oxide (SGO)-modified polysulfone-polyamide forward osmosis (FO) membrane for the reduction of fluoride ions (F^−^) in drinking water. The results of the authors’ study showed that the synthesized SGO-modified polysulfone-polyamide FO membrane had excellent reduction performance against the fluoride ions in the drinking water. Hence, SGO-modified polysulfone-polyamide FO membranes can be efficiently applied in drinking water purification processes [299].

High levels of chlorine and monochloramine in drinking water pose great danger to human health. Hence, their removal from drinking water is crucial. Foller, et al. [300] synthesized graphene oxide (GO) membranes for the removal of chlorine and chloramine from drinking water. The results of the authors’ study showed that the GO membrane successfully removed a high percentage of both chlorine and chloramine from drinking water. Hence, GO membranes are efficient candidates that can be applied in the removal of contaminants from drinking water [300]. Hasanzadeh, et al. [301] investigated the use of graphene oxide (GO) membranes in the removal of chromium (VI) ion from drinking water. The results of the authors’ study showed that GO membranes efficiently removed chromium (VI) ions from water. Hence, graphene oxide-based membranes are potential candidates that can be applied successfully in the removal of heavy metal ions from drinking water [301]. Tian, et al. [302] studied the use of highly permeable GO membranes for water purification. The results of the authors’ study showed that the synthesized membrane successfully removed more that 91% of Coomassie brilliant blue dye and greater than 95% of natural organic matter (humic acid and bovine serum albumin) from water. According to the results of this study, GO-based membranes are fully capable of removing pollutants from drinking water and can be applied efficiently in drinking water purification processes [302]. Koli, et al. [303] synthesized a thin-film composite (TFC) membrane over a highly permeable mixed matrix support ultra-filtration membrane (UF membrane) using sulfonic acid-functionalized graphene oxide (SGO) and polyethersulfone (PES) for the removal of contaminants from groundwater. The results of the authors’ study showed that the fabricated PES-SGO-TFC membrane removed 89% of Cr(VI), 99% of As(V), and 77% of fluoride that were present in the groundwater. In addition, the membrane showed more than 90% removal for monovalent fluoride ions that were found in the groundwater. Hence, membrane technology is very efficient in the removal of heavy metal ions from drinking water, and can be used in drinking water purification processes [303].

Although waterborne virus removal may be achieved using separation membrane technologies, such technologies remain largely inefficient at generating virus-free effluents due to the lack of anti-viral reactivity of conventional membrane materials required to deactivate viruses. Here, a stepwise approach towards the simultaneous filtration and disinfection of Human Coronavirus 229E (HCoV-229E) in water effluents is proposed by engineering dry-spun ultrafiltration carbon nanotube (CNT) membranes, coated with anti-viral SnO_2_ thin films via atomic layer deposition. The thickness and pore size of the engineered CNT membranes were fine-tuned by varying spinnable CNT sheets and their relative orientations on carbon nanofiber (CNF) porous supports to reach thicknesses less than 1 µm and pore sizes of around 28 nm. The nanoscale SnO_2_ coatings were found to further reduce the pore size down to ∼21 nm and provide more functional groups on the membrane surface to capture the viruses via size exclusion and electrostatic attractions. The synthesized CNT and SnO_2_-coated CNT membranes were shown to attain a viral removal efficiency above 6.7 log_10_ against the HCoV-229E virus with fast water permeance up to ∼4 × 10^3^ and 3.5 × 10^3^ L.m^−2^.h^−1^.bar^−1^, respectively. Such high performance was achieved by increasing the dry-spun CNT sheets up to 60 layers, orienting successive 30 CNT layers at 45°, and coating 40 nm SnO_2_ on the synthesized membranes. The current study provides an efficient scalable fabrication scheme to engineer flexible ultrafiltration CNT-based membranes for the cost-effective filtration and inactivation of waterborne viruses to outperform state-of-the-art ultrafiltration membranes [304].

To efficiently improve the performance of ultrafiltration (UF) membrane for drinking water treatment, carbon nanotubes (CNTs) and carbon nanofibers (CNFs) have been utilized as pre-deposited coating layers on the membrane surface. A comparative study between these two carbon nanomaterials for enhancing pollutant removal and mitigating membrane fouling induced by natural organic matter (NOM) was carried out. The surface morphologies were characterized by scanning electron microscopy, and the results indicated that the CNT coating layer was more dense and homogeneous with a smaller pore size than that of CNFs. The removal and antifouling performance of CNT/CNF-coated membranes were investigated with typical NOM, i.e., humic acid, bovine serum albumin, sodium alginate, as well as natural surface water. The results showed that the presence of coating layers was very effective in improving the rejection rate of NOM, among which CNTs exhibited significantly better performance than CNFs. The fouling control performance was influenced by the NOM fraction and coating mass (6–50 g/m^2^). Generally, the CNT coating layer was more efficient in alleviating both reversible and irreversible membrane fouling, while CNFs exhibited a limited effect on irreversible fouling control. Both pre-adsorption and size exclusion contributed to the rejection of membrane foulants, thus reducing the organics directly contacted with the underlying membrane. In natural surface water treatment, the pre-deposited coating layers significantly delayed the transition of fouling mechanisms from pore blocking to cake filtration. The experimental results were expected to illustrate the feasibility of pre-deposited CNT/CNF layers for enhancing membrane performance during drinking water treatment [305].

Arsenic contamination is threatening drinking water safety in many areas around the world. Among different arsenic species in water, arsenite or As(III) is the most difficult one to remove due to its neutrality in drinking water. Therefore, the development of novel materials is warranted for efficient As(III) removal. Herein, novel nanocomposite materials were facilely prepared by intercalating Cu nanoparticles into carboxylated/hydroxylated carbon nanotubes (CNT) deposited on top of a conventional polymeric membrane. The as-prepared membrane possessed high pure water permeability (4639–4854 L m^−2^ h^−1^ bar^−1^) and the capability to remove more than 90% of As(III) at transmembrane pressures below 0.01 bar, which is plausible for gravity-driven membrane (GDM) filtration applications. Compared to Fe-based materials reported in the literature, the Cu/CNT nanocomposite membranes exhibited robustness in removing As(III) at a pH range of 5–9 and in the presence of chloride, nitrate, bicarbonate, sulfate, and natural organic matter. A mechanical investigation suggests that the filtration process involves the direct adsorption of As(III) onto Cu/CNT composites. As(V) species were also produced due to the oxidation of As(III) by dissolved oxygen under the catalysis of Cu and adsorbed by Cu/CNT composites. Overall, the novel Cu/CNT nanocomposite membranes exhibit promising potential in As(III) removal from contaminated water, and are worthy of further investigation for their suitability in drinking water treatment [294].

The discharge of pharmaceuticals has several negative impacts on nature and poses severe danger to human health. Due to the strong resistance of certain pharmaceutical product (PP) wastes to biodegradation, physical-based separation technologies, like forward osmosis (FO), have been developed for the elimination of these unwanted products. Lee, et al. [306] studied the application of carbon nanotube (CNT)-based forward osmosis membranes (FO) in the elimination of paracetamol from water. The results of the authors’ study showed that the synthesized CNT-based membrane successfully eliminated up to 99.7% of the paracetamol present in water. Hence, FO membranes modified with CNTs are potential candidates for the purification of drinking water from pharmaceutical products [306]. Feng, et al. [307] investigated the effect of depositing TiO_2_ nanoparticles on CNT membranes on the removal of chromium ions from water. The results of the authors’ study showed that the TiO_2_-deposited CNT membranes exhibited high removal efficiencies of Cr and Cr(VI) with values of 92.1% and 93.3%, respectively. Hence, TiO_2_-deposited CNT membranes are potential candidates for the removal of heavy metal ions from drinking water [307]. Yang, et al. [308] investigated the use of CNT-based membrane in the removal of several dyes (rhodamine B, methylene blue, eosin Y, and acid orange 7) from water. The results of the authors’ study showed that the CNT-based membrane exhibited remarkable adsorption capacities for all the dyes present in the water, with adsorption capacity values of 181 and 247 mg/g for rhodamine B and methylene blue dyes, respectively. Therefore, CNT-based membranes are efficient candidates that can be applied in the removal of dyes from drinking water [308]. He, et al. [309] studied the application of multi-walled carbon nanotube (MWCNT) membranes in the removal of humic substances (HSs) from natural water. The results of the authors’ study revealed that the MWCNT membrane exhibited a high removal of HSs, with a removal efficiency of greater than 85%, from natural water. Hence, MWCNT membranes have great potential in the removal of contaminants from drinking water systems [309]. Ali, et al. [310] studied the use of functionalized-MWCNT (f-MWCNT) membranes in the removal of zinc ions (Zn^2+^) from synthetic water and real wastewater effluents. The results of the authors’ study showed that f-CNT membranes successfully removed up to 98% and 70% of the Zn^2+^ present in synthetic water and real wastewater effluents. Based on the results of this study, MWCNT membranes are efficient candidates that can be applied in the removal of heavy metal ions from drinking water [310]. According to all of the above studies, membrane technologies are very efficient in the removal of several types of pollutants from water and can be applied in drinking water purification processes. Table 15 shows a summary of nanomaterial-based membrane applications in water purification.

## 6. Conclusions

The utilization of nanomaterials in water purification technologies offers cutting-edge techniques for tackling the global water contamination crisis, providing advanced solutions with higher efficiency and specificity than conventional water treatment methods. This review paper focused on several key nanomaterials for water purification including graphene and its derivatives, carbon nanotubes (CNTs), titanium dioxide nanoparticles (TiO_2_ NPs), zinc oxide nanoparticles (ZnO NPs), silver nanoparticles (Ag NPs), iron nanoparticles (Fe NPs), and membrane-based nanomaterials. Each nanomaterial has distinct properties that make them highly effective in the removal of various pollutants from water.

Graphene and its derivatives (graphene oxide (GO) and reduced graphene oxide (rGO)) have shown great promise in the removal of contaminants for water purification owing to their high surface area, mechanical strength, and versatile surface chemistry. Furthermore, carbon nanotubes (CNTs) with their hollow cylindrical structure and large surface area have shown great performance in the adsorption of organic pollutants and heavy metals from water. In addition, titanium dioxide nanoparticles (TiO_2_ NPs) with their exquisite photocatalytic properties are able to degrade organic pollutants under UV or visible light irradiation. Furthermore, zinc oxide nanoparticles (ZnO NPs) exhibit high photocatalytic and antimicrobial properties, making them promising candidates for water purification systems. Furthermore, silver nanoparticles (Ag NPs) exhibit strong antimicrobial properties, making them capable of inactivating a broad spectrum of pathogens, including bacteria, viruses, and fungi. Moreover, iron nanoparticles (Fe NPs) have been extensively applied in the removal of heavy metals and organic pollutants from water via adsorption and redox reactions. Furthermore, nanomaterials incorporated into membranes have significantly improved the performance of filtration technologies. The integration of nanomaterials like TiO_2_, ZnO, or graphene-based materials into membranes has greatly enhanced membrane fouling resistance, mechanical strength, and selectivity for contaminants.

Despite the remarkable promising results of nanomaterial application in water purification, challenges remain in the large-scale application of these nanomaterials. Several issues including nanoparticle release into the environment, high production costs, scalability, and long-term stability of nanomaterials must be overcome. Additionally, the permissible limits for the release of these nanomaterials into the environment must be comprehensively evaluated to avoid any potential harm that could impact the environment or human health.

The future of nanomaterial-based water purification lies in addressing the existing limitations on a large-scale industrial level. The next generation of nanomaterials should be synthesized using green and sustainable production methods. For example, using biological methods to produce nanoparticles offers a sustainable alternative to chemical synthesis, with a lower carbon footprint. In addition, the use of waste to produce nanomaterials for water purification holds great promise. This approach not only reduces the overall carbon footprint associated with the production of nanomaterials, but it also considers waste as a valuable resource. Future research should also focus on the functionalization of nanomaterials to improve their selectivity for the removal of specific contaminants. This can be achieved by using functional groups, ligands, or surface coatings to increase the adsorption capacity of the nanomaterial for targeted pollutants. In addition, the development of hybrid nanomaterials that combine the properties of several nanomaterials, such as graphene–TiO_2_ composites, will increase the removal efficiency of contaminants from water. Finally, the goal of future research must be focused on narrowing the existing gap between laboratory research and real-life applications by conducting large-scale projects that utilize several nanomaterials for water purification with extensive field tests. In conclusion, the future of water purification using nanomaterials is promising, but requires a great effort to overcome technical, environmental, and regulatory challenges.

## Figures and Tables

**Table 1:** MCLG allowed by the EPA for some common organic contaminants that are found in drinking water and their potential health side effects in humans.

Organic Contaminant	US EPA Maximum Contaminants Level Goals Allowed (MCLG) (mg/L)	Potential Health Adverse Effects
Benzene	0	Increased risk of cancer
Carbon tetrachloride	0	Liver problems and increased risk of cancer
Chlorobenzene	0.1	Liver and kidney problems
Acrylamide	0	Nervous system problems and increased risk of cancer
Carbofuran	0.04	Issues with blood, nervous system, and reproductive system
Alachlor	0	Eye, liver, kidney, and spleen problems. In addition, it increases the risk of developing cancer
1,2-Dichloroethane	0	High risk of developing cancer
Dichloromethane	0	Increased risk of liver cancer
1,2-Dichloropropane	0	High risk of cancer

**Table 2:** MCLG allowed by the EPA for some common inorganic contaminants that are found in drinking water and their potential health side effects in humans.

Inorganic Contaminant	US EPA Maximum Contaminants Level Goals Allowed (MCLG) (mg/L)	Potential Health Adverse Effects
Arsenic	0	High risk of developing skin, bladder, and lung cancers
Antimony	0.006	Nausea, vomiting, and diarrhea
Barium	2	Cardiovascular and kidney diseases, metabolic issues, and neurological problems
Cadmium	0.005	Kidney damage
Beryllium	0.004	Intestinal lesions
Chromium	0.1	Allergic dermatitis, liver damage, and kidney problems
Copper	1.3	Liver damage, diarrhea, stomach cramps, nausea, vomiting, and kidney disease.
Cyanide	0.2	Nervous system damage and thyroid problems
Fluoride	4	Bone disease and teeth problems
Mercury	0.002	Kidney damage
Lead	0	Delay in the physical and mental development of newborns and children. Adults will also experience problems with their kidneys and high blood pressure.
Selenium	0.05	Skin and tooth discoloration, pathological deformation and nail loss, hair loss, severe tooth decay, and lack of mental alertness
Nitrate	10	For newborn, babies high nitrate concentrations in drinking water may lead to shortness of breath and blue-baby syndrome

**Table 3:** MCLG allowed by the EPA for some common microorganisms that are found in drinking water and their potential health side effects in humans.

Microorganisms	US EPA Maximum Contaminants Level Goals Allowed (MCLG)(mg/L)	Potential Health Adverse Effects
Cryptosporidium	0	Gastrointestinal illness
Total coliforms (fecal coliform and *E.coli*)	0	Gastrointestinal illness
*Giardia lamblia*	0	Gastrointestinal illness
Viruses (enteric)	0	Gastrointestinal illness
*Legionella*	0	Legionnaire’s disease

**Table 4:** MCLG allowed by the EPA for some common disinfection byproducts (DBPs) that are found in drinking water and their potential health side effects in humans.

Disinfection Byproducts (DBPs)	US EPA Maximum Contaminants Level Goals Allowed (MCLG) (mg/L)	Potential Health Adverse Effects
Chlorite	0.8	Irritation in the mouth, esophagus, or stomach
Haloacetic acids (HAA5)	n/a	Carcinogenic effects
Bromate	0	Carcinogenic effects
Total trihalomethanes (TTHMs)	n/a	Increased risk of developing bladder cancer, rectal cancer colon cancer, and adverse developmental and reproductive effects during pregnancy

**Table 5:** MRDL allowed set by the EPA for some commonly applied disinfectants in drinking water treatment applications, along with their potential health side effects.

Disinfection Byproducts (DBPs)	US EPA Maximum Residual Disinfectant Level (MRDLG) (mg/L)	Potential Health Adverse Effects
Chlorine (Cl_2_)	4	Irritation in the mouth, esophagus, or stomach
Chloramines (Cl_2_)	4	Carcinogenic effects
Chlorine dioxide (ClO_2_)	0.8	Carcinogenic effects
Total trihalomethanes (TTHMs)	n/a	Increased risk of developing bladder cancer, rectal cancer colon cancer, and adverse developmental and reproductive effects during pregnancy

**Table 6:** MCLG allowed by the EPA for some common radionuclides that are found in drinking water and their potential health side effects in humans.

Radionuclides	US EPA Maximum Contaminants Level Goals Allowed (MCLG) (mg/L)	Potential Health Adverse Effects
Alpha particles	0	High risk of developing cancer
Beta particles and photon emitters	0	High risk of developing cancer
Uranium	0	High risk of developing cancer and kidney failure
Radium 226 and Radium 228	0	High risk of developing cancer

**Table 7:** Unique properties of nanomaterials and their applications in the drinking water purification process.

Properties of Nanomaterials	Application in Drinking Water Purification Process
Large Surface Area	Nanomaterials are characterized by having a remarkable large surface area-to-volume ratio owing to their nanoscale dimensions. This unique property allows the nanomaterials to have an increased contact with the pollutants found in drinking water, making them an efficient adsorbents and catalysts.
High Reactivity	The high chemical and physical reactivity of nanomaterials is particularly beneficial in the catalytic processes, where the contaminants in drinking water are broken down.
Selective Adsorption	The surface of nanomaterials can be specially engineered to selectively adsorb certain types of pollutants in drinking water. Their surface chemistry can be modified easily to target particular contaminants, making them a versatile substance that is capable of removing a wide range of pollutants from drinking water.
High Mechanical Strength	Certain types of nanomaterials, like nanocomposites, have a high mechanical strength and durability. This unique property is very useful in the production of reinforced water infrastructure components that prevents corrosion and leakage.
Improved Optical Properties	Specific types of nanomaterials, like quantum dots, have distinguishable optical properties that can be used in sensing and monitoring water quality. They can change their fluorescence or absorbance properties in the presence of certain types of contaminants.
Size-Dependent Properties	The nanomaterial properties depend mainly on their size, and changing the size of nanomaterials completely alters their properties. Therefore, by controlling nanomaterial size, their properties can be modified to suit different water treatment applications.
Stability and Regeneration	Certain nanomaterials are highly stable and can be regenerated. This reduces the overall cost of the drinking water purification process.
Photocatalytic Activity	Some nanomaterials, including titanium dioxide and zinc oxide nanoparticles, have a high photocatalytic activity, which means that they are capable of breaking down organic pollutants and inactivate microorganisms in the presence of ultraviolet (UV) light.
Antimicrobial Properties	Some nanomaterials, like silver nanoparticles, have antimicrobial properties that helps in disinfecting drinking water by preventing the growth of harmful microorganisms.

**Table 8:** Summary of graphene-based adsorbents’ application in the water purification process.

Graphene-Based Adsorbent	Water Contaminant	Adsorption Capacity (mg/g)	Reference
GO	Methylene blue (MB)	598.8	[59]
SiO_2_/graphene	Lead (II)	113.6	[60]
Chitosan-doped GO (CSGO aerogel)	Methyl orange (MO) and amido black 10B (AB 10B)	686.89 and 573.47	[61]
Magnetic cellulose GO (MCGO)	Methylene blue (MB)	70.03	[62]
*N*-phenylthiourea graphene oxide (GO-NPT)	Methyl orange (MO)	141.467	[63]
Konjac glucomannan (KGM/GO Hydrogel)	Methylene blue (MB) and methyl orange (MO)	133.67 and 93.5	[64]
Single-layered GO sheet	Basic red 12 (BR 12) and methyl orange (MO)	63.69 and 16.83	[65]
3D GO-NiFe-layered double hydroxide (GO-NiFe LDH)	Congo red (CR) and methyl orange (MO)	450 and 403	[66]
Magnetic GO (GO-MNP)	Methylene blue (MB)	1666.67	[67]
Graphene modified by cetyltrimethylammonium bromide	Cr (VI)	21.57	[68]
Graphene	Antimony (III)	8.056	[69]
Activated carbon (AC), GO, carbon nanotubes (CNTs)	Methylene blue (MB)	270.27, 243.9 and 188.68	[70]
Reduced GO/TiO_2_ (MrGO/TiO_2_ and rGO/TiO_2_)	Methylene blue (MB)	845.6 and 467.6	[71]
Graphene nanosheets (GNSs)	Lead (II)	35.46	[72]
Graphite, graphite oxide, and graphene sheet	Fe^2+^ and Co^2+^	299.3 and 370	
GO/Fe_3_O_4_	Cu(II)	18.3	[73]
GO/silica/Fe_3_O_4_	Pb and Cd (II)	333.3 and 166.7	[74]
GO/Fe_3_O_4_/sulfanilic acid	Cd (II)	55.4	[75]
GO-MnFe_2_O_4_	Pb(II)	673	[76]
rGo/COFe_2_O_4_	Pb(II)	299.4	[77]
GO/Mn-doped Fe(III)oxide	Cd and Cu(II)	87.2 and 129.7	[78]
GO/Fe_3_O_4_/sulfanilic acid	Cu(II)	50.7 and 56.8	[79]
GO/Fe_3_O_4_	Cu, Pb, and Cd	23.1, 38.5 and 4.4	[80]
Graphene oxide (GO)	Pb(II)	35.6	[81]
TiO_2_/GO	Pb(II)	65.6	[82]
GO-EDTA	Pb(II)	479	[82]
Graphene nanosheets (GNs)	Pb(II)	22.4	[72]
GNs-500	Pb(II)	35.2	[72]
MnO_2_/GNs	Hg (II)	10.8	[83]
Polyethersulfone/GO	MB	62.50	[84]
Fe_3_O_4_/GO hybrids	MB, NR	167.2, 171.3	[85]
GO sponge	MB, MV	397, 467	[86]
Fe_3_O_4_–GNs	MB, CR	45.3, 33.7	[87]
GO	MB	351	[88]
GNs	MB	154–204	[89]
GO	Tetracycline	313.48	[90]
GO	Oxytetracycline	212.31	[90]
GO	Doxycycline	398.40	[90]
F–GO with MNPs	Oxytetracycline	45.0	[91]
F–GO with MNPs	Tetracycline	39.1	[91]
F–GO with MNPs	Chlortetracycline	42.6	[91]
F–GO with MNPs	Doxycycline	35.5	[91]
TiO_2_-graphene sponge	Tetracycline	1805	[92]
GO	Tetracycline	381.77	[93]
Single-layer GO	Ciprofloxacin	379	[94]
KOH-activated graphene	Ciprofloxacin	194.6	[95]
Porous graphene hydrogel	Ciprofloxacin	235.6	[96]

**Table 9:** Summary of CNT-based adsorbent applications in the water purification process.

CNT Based-Adsorbent	Water Contaminant	Adsorption Capacity (mg/g)	Reference
CNTs	Pb^2+^	17.44	[122]
CNTs (HNO_3_)	Pb^2+^	49.95	[123]
MWCNTs	Ni^2+^	7.53	[124]
SWCNTs	Ni^2+^	9.22	[124]
MWCNTs (HNO_3_)	Pb^2+^	97.08	[125]
Alkali-activated MWCNTs	Methylene blue	399	[126]
Untreated MWCNTs	Methylene blue	59.7	[127]
Untreated SWCNTs	Reactive red 120 (RR −120)	426.49	[128]
Oxidized SWCNTS	Basic red 46 (BR 46)	49.45	[129]
Untreated MWCNTs	Tetracycline (TC)	269.54	[130]
MWCNTs	Olaquindox	99.7%	[131]
SWCNTs	4-Chloro-2-nitrophenol	1.44	[132]
MWCNTs	4-Chloro-2-nitrophenol	4.42	[132]
Untreated SWCNTs	Dissolved organic matter (DOM)	26.1–20.8	[133]
KOH-activated MWCNTs	Toluene, ethylbenzene, m-xylene	87.12, 322.05, 247.83	[134]
MWCNTs	Methyl orange	52.86	[135]
Chitosan/Fe_2_O_3_/MWCNTs	Methyl orange	66.90	[136]
Calcium alginate/MWCNTs	Methyl orange	12.5	[136]
MWCNTs	Tetracycline	192.7	[137]
CNTs-C@Fe-chitosan composite	Tetracycline	104	[138]
Single, double, and multi-walled carbon nanotubes	Oxytetracycline	554, 507, 391	[139]
Single, double, and multi-walled carbon nanotubes	Ciprofloxacin	933.8, 901.2, 651.4	[139]
MWCNTs/CoFe_2_O_4_	Sulfamethoxazole	6.98	[140]
Pristine and hydroxylated MWCNTs	Sulfamethazine	24.78, 13.31	[141]
Carboxylated multi-walled carbon nanotubes	Norfloxacin	90.3	[142]

**Table 10:** Summary of TiO_2_-nanoparticle-based material applications in the water purification process.

TiO_2_ Nanoparticle-Based Material	Water Contaminant	Main Research Results	References
Porous TiO_2_ nanotubes (PTNTs)	Congo red dye (CR), Cr(VI)	High adsorption capacity of Cr(VI) from water reaching 540 mg/g. High photocatalytic degradation of CR dye.	[164]
HfO_2_-doped TiO_2_ (HfO_2_/TiO_2_)	Methyl orange (MO), methyl blue (MB), cresol red (CR), thymol blue (TB), and solochrome black (SB).	High photocatalytic degradation of MB, MO, and CR, SB, and TB dyes was achieved. The highest degradation efficiency reached was 90% for MB dye.	[165]
Cu-modified TiO_2_	*E. coli*, antibiotics	A total of 75% of total organic carbon was eliminated from water within 6 h. All antibiotics were degraded within 30 min.	[166]
Poly methyl methacrylate/ TiO_2_ (TiO_2_/PMMA)	Methylene blue (MB)	High percentage removal of MB dye was achieved.	[167]
AgInS_2_/TiO_2_ nanobelts	*E. coli*, Cr(VI), bisphenol A (BPA)	A total of 88.5% of BPA was effectively degraded in water. A total of 100.0% of Cr(VI) was effectively eliminated from water. High percentage removal of *E. coli* bacteria was achieved	[168]
Anatase-phase TiO_2_ nanoparticles	Rhodamine B (RhB)	A total of 90% degradation of RhB dye was achieved within 80 min from water.	[169]
TiO_2_-rGO nanocomposites	Pesticides (methomyl, isoproturon, pyrimethanil, alachlor)	A total of 100% pesticide removal was achieved.	[170]
Fe_3_O_4_–TiO_2_/rGO	Atrazine	A total of 100% degradation of atrazine was achieved.	[171]
TiO_2_	The herbicide 2,4D and insecticide imidacloprid	A total of 100% removal of pesticides found in water was achieved. A total of 35% degradation of imidacloprid was achieved.	[172]
TiO_2_ APTES (3-Aminopropyl)triethoxysilane)	*E. coli*	The use of APTES enhanced antibacterial properties. Most of the *E.coli* bacteria was removed from water.	[173]
Sulphur-doped TiO_2_	Organophosphorus pesticides (quinalphos and mono-crotophos)	High percentage degradation of quinalphos reaching 98%. High percentage degradation of monocrotophos reaching 95%.	[174]
MoS_2_/TiO_2_ nanotube arrays	Ibuprofen, antibiotics, methicillin-resistant Staphylococcus aureus (MRSA), *E. coli*	Most of the bacteria was successfully removed from water. Great disinfecting properties. High water disinfection efficiency reaching 98.5%.	[175]
TiO_2_/Au/Fe_2_O_3_	*S. typhimurium*, 2,4 dichlorophenol	High photocatalytic performance.	[176]
Ag@TiO_2_/NPs	*E. coli*	High water disinfection activity. High removal of *E.coli* from water.	[177]
Ag/TiO_2_ nanofibers	Phenol	High percentage removal of phenol reaching 82.65% was achieved.	[178]
ZnO–TiO_2_	Methylene blue (MB)	High photodegradation rate of MB dye.	[179]
Ag@ TiO_2_ NPs	*E. coli*	High percentage removal of *E.coli* bacteria was achieved.	[180]
Nitrogen-doped TiO_2_	Ciprofloxacin	A 90% removal rate of ciprofloxacin was achieved within 90 min.	[181]
Nitrogen-doped TiO_2_ fibers	Methylene blue (MB)	High photocatalytic activity. High percentage degradation of MB dye was achieved.	[182]
Sulphur-doped TiO_2_	Diclofenac	High percentage degradation of diclofenac was achieved reaching 93% within 4 h.	[183]

**Table 11:** Summary of ZnO nanoparticle-based material applications in the water purification process.

ZnO Nanoparticle-Based Material	Water Contaminant	Research Main Results	References
ZnO@silica	Crystal violet dye (CV)	High percentage removal of CV dye was reached with a value of 98%.	[204]
Capped ZnO	RR141 (azo dye) and the ofloxacin antibiotic (OFL)	Up to 100% and 98% removal of RR141 and OFL was achieved.	[205]
ZnO/Ni microtubes	Textile dyes	High photocatalytic degradation of textile dyes from water with a value reaching 91.21% was achieved.	[206]
ZnO/cellulose ZnO/polyester	*E. coli*	ZnO/polyester resulted in a 15% higher disinfection efficiency against *E.coli* bacteria compared to ZnO/cellulose.	[207]
ZnO nanorods	Methylene blue (MB) dye	High percentage removal of MB dye from water with a value reaching 94% was achieved.	[208]
ZnO NPs	Methylene blue (MB) dye	High photocatalytic degradation efficiency of MB dye was reached.	[209]
ZnO/PALFs	Congo red dye (CR)	Up to 95% photocatalytic degradation efficiency of CR dye was achieved.	[210]
Nano-ZnO	*E. coli*	High photo-activity and bactericidal activity against *E.coli* bacteria was achieved.	[211]
ZnO NPs	Glyphosate-based herbicides	A total of 90% of the herbicide was removed.	[212]
ZnO/mullite membranes ZnO/alumina membranes	*S. aureus*	Up to 35% disinfection efficiency for mullite membranes was achieved. Up to 45% disinfection efficiency for alumina membranes was reached. Greater than 99% disinfection efficiency for both ZnO/mullite and ZnO/alumina membranes was successfully targeted.	[213]
ZnO/AAO nano membranes	*E. coli*	High percentage removal of *E. coli* bacteria was reached.	[214]
Red phosphorous/ZnO (RP/ZnO)	*S. aureus*, *E. coli*	Remarkable antibacterial efficiency with values of 99.96% and 99.97% against *S. aureus* and *E. coli*, respectively.	[215]
ZnO/CdS	The ofloxacin antibiotic (OFL) and RR141 (azo dye)	A total of 80% and 73% removal of RR141 and OFL was reached.	[216]
ZnO/La_2_O_3_/NiO NPs	MB	High photocatalytic degradation efficiency with a value reaching 98% for MB dye.	[217]
ZnO/SnO_2_	The pesticide triclopyr	High percentage degradation of the pesticide triclopyr was reached.	[218]
g-C_3_N_4_/NiO/ZnO/Fe_3_O_4_	Esomeprazole	A 95.05% degradation efficiency was reached within 70 min.	[219]
ZnO@ZnS core@shell Cu cables	Methylene blue, p-nitrophenol, and rhodamine B	Remarkable photocatalytic degradation with a value greater than 99%.	[220]
ZnO–ZnTe	MB, azo dye	Efficient removal of dyes from water was achieved with a value of 91% for MB dye.	[221]
Bi_2_WO_6_/ZnO	RhB	Up to 99% photodegradation efficiency for RhB dye was reached.	[222]
g-C_3_N_4_/Mn–ZnO	MB, Gram-positive and negative bacteria	Remarkable degradation efficiency for MB dye was reached. Furthermore, excellent antibacterial activity was achieved against Gram-positive and negative bacteria.	[223]
ZnO–TiO_2_	MB	High photodegradation of MB dye was achieved.	[179]
Pt nanoparticles/polypyrrole-carbon black/ZnO (Pt@PPy-C/ZnO)	MB dye and the linezolid antibiotic	High percentage removal of linezolid with a value of 94.0% was reached within 40 min, and most of the MB dye was successfully degraded.	[224]
Co/ZnO thin film Cu/ZnO thin film	MB	Co/ZnO and Cu/ZnO resulted in a 62.6% and 42.5% degradation of MB dye, respectively.	[225]
Nd-ZnO NP	MB	Up to 98% degradation efficiency was reached for MB dye.	[226]
La-doped ZnO NP and bare ZnO NP	Paracetamol	La-doped ZnO nanoparticles exhibited higher photocatalytic activity compared to bare ZnO NPs. Most of the paracetamol was successfully removed from water.	[227]

**Table 12:** Summary of Ag nanoparticle-based material applications in the water purification process.

Ag Nanoparticle-Based Material	Water Contaminant	Research Main Results	References
Ag-doped Mg_4_Ta_2_O_9_ NPs	The herbicide atrazine, rhodamine B, methyl orange (MO)	High photocatalytic degradation efficiency of dyes was achieved.	[245]
Ag NPs in cellulose polymer paper	Organic pollutants (4-NP, 2-nitrophenol (2-NP), 2- nitroaniline (2-NA), TNP)	Up to 99% reduction in contaminants was achieved.	[246]
Ag NPs/holocellulosenanofibrils (Ag NPs/HCNFs)	Methylene blue (MB)	98% degradation of MB dye was achieved.	[247]
Silver-decorated magnetic Fe_3_O_4_-deposited thioglycolic acid-modified polypyrrole nanocomposite (Fe_3_O_4_@PPy- MAA/Ag)	4-NP, methylene blue (MB), methyl orange (MO)	High percentage degradation of dyes was achieved with a value of approximately 100% degradation for 4-NP.	[248]
Ag NPs capped on novel nanocomposite 2- hydroxypropylβ-cyclodextrin/alginate	4-NP, methyl orange (MO), rhodamine B	Remarkable dye degradation efficiency was achieved.	[249]
TiO_2_@CNTs/Ag NP/surfactant (C10) nanocomposite	Methylene blue (MB)	A 100% MB dye degradation was achieved within 180 min.	[250]
Ag NPs	Textile dyes (reactive green 19A, reactive blue 59, reactive red 120, reactive red 141, and reactive red 2)	Up to 50% of the textile dyes were efficiently removed from water.	[251]
AgNPs@ZIF-8 composite	Methylene blue (MB) and Congo red (CR)	97.25% and 100% removal of MB and CR dyes was achieved, respectively.	[252]
Iron-silver core-shell NPs (FeO/Ag NPs)	Aniline blue dye	High aniline blue dye degradation efficiency with a value reaching 90%.	[253]
Ag/BiVO_4_ photocatalyst	2,4-dichlorophenoxyacetic acid	Up to 90% degradation was achieved.	[254]
GO nanosheets impregnated with Ag NPs	*E. coli*, *S. aureus*	Up to 100%, and 87.6% reduction of *E. Coli* and *S. aureus* was achieved	[255]
Ag NPs	4- NP, methyl orange (MO), and rhodamine B	Excellent dye degradation efficiency.	[256]
GO-AgNP composite onto cellulose acetate membrane	*E. coli*	Up 86% of the *E. coli* bacteria were inactivated within 2 h.	[257]
Hierarchical Cu_2_O nanowires covered by AgNP-doped carbon layer on Cu foam (C/Cu_2_OAgNPs)	*E. coli*, *S. aureus*	High bactericidal effect with a value greater than 99% was achieved.	[258]
GO-Ag nanocomposite	*E. coli*, *S. aureus*	High bactericidal effect was achieved.	[259]
Ag NP-decorated cellulose nanofibrous membrane	Coliform bacteria	Almost complete removal of bacteria.	[260]
Ag NPs loaded on tannic acid-impregnated cellulosic membrane	*E. coli*	Up to 97% reduction percentage of the *E.coli* bacteria was achieved.	[261]

**Table 13:** Summary of iron nanoparticle-based material applications in the water purification process.

Iron Nanoparticle (Fe NPs)-Based Material	Water Contaminant	Adsorption Capacity (mg/g)	Removal Efficiency (%)	References
A mixture of α-Fe_2_O_3_ and Fe_3_O_4_ modified with sugarcane bagasse biochar	Cr (VI) ions	24.48	92.07	[272]
γ-Fe_2_O_3_	Arsenic	12.74	95	[273]
Mixture of γ-Fe_2_O_3_ and Fe_3_O_4_	Cu	11.12	90	[274]
Mixture of γ-Fe_2_O_3_ and Fe_3_O_4_	Ni	9.44	95	[274]
A mixture of Fe_2_O_3_, FeO, and Fe_3_O_4_ modified with rice straw biochar	Crystal violet	111.48	97.23	[275]
Mixture of γ-Fe_2_O_3_ and Fe_3_O_4_	Cd	19.15	90	[274]
Fe_3_O_4_ modified with carboxymethyl- β-cyclodextrin polymer	Lead (II) ions	64.2	98	[276]
Fe_3_O_4_ modified with PAC from pistachio waste	Cu (II)	23.61	98	[277]
Fe_3_O_4_ functionalized with ethylenediamine	Chromium	81.5	96	[278]
Fe_3_O_4_	Phosphate	8.79	88	[279]
Fe_3_O_4_ modified with sugarcane bagasse biochar	Cr (VI) ions	71.02	–	[280]
Fe_3_O_4_	Cadmium	48.82	100	[281]
Fe_3_O_4_	Lead (II) ions	120.48	–	[282]
Fe_3_O_4_	Boron	8.44	84	[283]
Fe_3_O_4_	Arsenic	8.31	78	[284]
α-Fe_2_O_3_ modified with sodium alginate	Lead (II) ions	564	–	[285]
Fe_3_O_4_ functionalized with chitosan	Copper (II) ions	109.89	92	[286]
Fe_3_O_4_	Arsenic	90	–	[287]

**Table 14:** Different types of membranes and their applications in the drinking water purification process.

Membrane Type	Pore Size	Application in Water Purification	References
Microfiltration (MF) membrane	0.1 to 10 μm	Removes suspended solids, bacteria, and protozoa.	[288]
Ultrafiltration (UF) membrane	0.001 to 0.1 μm	Removes viruses, colloids, and macromolecules including proteins and sugars.	[289]
Nanofiltration (NF) membrane	0.001 μm	Removes dissolved organic compounds, divalent ions, and pharmaceuticals.	[290]
Reverse osmosis (RO) membrane	0.0001 μm	Removes dissolved salts, including inorganic ions, organic compounds, and microorganisms.	[291]
Electrodialysis (ED) membrane	10 and 100 Å	ED membranes use an electrical current to move ions across a selective membrane. This ion diffusion through the membrane leads to the removal of dissolved salts from water.	[292]

**Table 15:** Summary of nanomaterial-based membrane applications in the water purification process.

Membrane Support Layer	Nanomaterial Used in Membrane Synthesis	Membrane Type	Water Contaminant	Removal Efficiency (%)	References
α-Al_2_O_3_	TiO_2_	UF	Rhodamine removal	>91	[311]
Bentonite	TiO_2_	UF	Anionic dyes (direct red 80 and acid orange 74) and a cationic dye (methylene blue)	98% for direct red 80 85% for acid orange 74 94% for methylene blue	[312]
Al_2_O_3_	TiO_2_	-	Organic dye	95	[313]
Bentonite	TiO_2_	UF	Direct red 80	99	[314]
Al_2_O_3_	TiO_2_	NF	Degradation of diuron/chlorfenvinphos	95% for diuron and 78% for chlorfenvinphos	[315]
Clay	TiO_2_	UF	Alizarin red dye	99	[316]
Al_2_O_3_/ZrO_2_	Yttria- stabilized	NF	Dye	>98	[317]
CuO	TiO_2_	UF	Ciprofloxacin removal	99.5	[318]
Al_2_O_3_	TiO_2_	UF	Natural organic matter (NOM; humic acid and tannic acid), pharmaceuticals (ibuprofen and sulfamethoxazole), and inorganic salts (NaCl, Na_2_SO_4_, CaCl_2_, and CaSO_4_).	93.5% for NOM 51.0% for pharmaceuticals 31.4% for inorganic salts	[319]
ZrO_2_	TiO_2_	NF	Co^2+^, Sr^2+^, and Cs^+^	99.6% for Co^2+^ 99.2% for Sr^2+^ 75.5% for Cs^+^	[320]
Rice husk	SiO_2_	UF	Methylene blue dye	99	[321]
Al_2_O_3_	SiO_2_	UF	Copper, cadmium, and silver	99	[322]
α-Al_2_O_3_	SiO_2_	NF	Divalent ions	–	[323]
TiO_2_-GO	γ-Al_2_O_3_	UF	Methyl orange and methylene blue	92	[324]
SiO_2_	MgO	MF	Tetracycline	99.7	[325]
Clay	ZrO_2_	UF	Silver	45	[326]
Ag/TiO_2_/hydroxya patite	α-Al_2_O_3_	MF	Humic acid	88.3	[327]
Al_2_O_3_	ZrO_2_	NF	Uranium	91	[328]
Al_2_O_3_	CuO	UF	Chromium (VI)	88.8	[329]
SiO_2_-TiO	SiC	-	Methylene blue removal	72	[330]
SiC	g-C_3_N_4_	-	Organic contaminants	100	[331]

## Data Availability

No new data were created or analyzed in this study.
